# Chemotherapy driven alterations in NK cell receptors and ligands in high grade serous ovarian cancer

**DOI:** 10.3389/fimmu.2026.1765987

**Published:** 2026-03-31

**Authors:** Pavan Kumar, Samruddhi Ranmale, Sanket Mehta, Hemant Tongaonkar, Vashishth Maniar, Jayanti Mania-Pramanik

**Affiliations:** 1 Infectious Diseases Biology, ICMR-National Institute for Research in Reproductive and Child Health, Mumbai, India; 2 Saifee Hospital, Mumbai, India; 3 P. D. Hinduja National Hospital and Medical Research Centre, Mumbai, India

**Keywords:** chemotherapy, high-grade serous ovarian cancer, immune modulation, ligands, NCR receptor

## Abstract

**Introduction:**

Combination approaches are being explored to improve immunotherapy efficacy, yet the immunomodulatory effects of chemotherapy on NK cell receptors and their ligands remain unexplored in high-grade serous ovarian cancer (HGSOC). Therefore, understanding chemotherapy-induced immune modulation is essential in HGSOC.

**Methods:**

Immune profiling was conducted on clinical specimens from 33 chemo-naïve patients undergoing primary debulking surgery (PDS), 57 chemotherapy-treated patients undergoing interval debulking surgery (IDS), and 17 patients in the IDS group were followed during chemotherapy cycles. Immune profiling was carried out using flow cytometry, Procartaplex immunoassay, and ELISA. Blood samples were collected from 50 age- and gender-matched healthy participants for comparison.

**Results:**

Primary investigation on follow-up patients reveals chemotherapy-mediated immune modulation on NK cell subsets. This was further validated in the chemo-treated surgical cohort. The Natural Cytotoxicity Receptors (NCRs) were downregulated on NK cells in chemo-naïve surgical cohorts. The NCR group of receptors was normalized to a level comparable to that in healthy controls in the chemotherapy-treated surgical cohort, due to reduced soluble ligands. Surface MICA expression was also increased (p = 0.0466) on EpCAM+ cells in the chemo-treated surgical cohort compared to the chemo-naïve surgical cohort, while NKG2D+ immune cells were reduced in both surgical cohorts compared to healthy controls. Moreover, proinflammatory cytokines IL-2 (p = 0.0001) and TNF-α (p = 0.0442) were reduced in the chemotherapy-treated surgical cohort, while intracellular levels of dual perforin and granzyme were elevated in the chemo-treated group, which may enhance cytolytic potential. High surface expressions of HLA-E, MIC-B, and LLT-1 ligands were associated with improved progression-free survival.

**Conclusions:**

Our findings highlight the broad immunomodulatory effects of chemotherapy on NK cell receptors, ligands, and cytokines, which may offer insights for combination therapies in HGSOC.

## 1 Introduction

Ovarian cancer presents significant mortality among gynecological cancers, due to late-stage diagnosis with an aggressive phenotype (1). The common treatment modality for ovarian cancer is debulking surgery to reduce the disease burden, followed by platinum-based chemotherapy or vice versa (2, 3). However, the prognosis of ovarian cancer varies even considering common factors such as stage, grade, and response to therapy. This disparity in the outcome of the disease may be driven by host characteristics. The host immune system is one such factor that has been proven to change the course of the disease (4, 5). Evidence shows that tumors can induce the onset of immune reactions (6), which may play a crucial role in eliminating neoplastic cells. However, ovarian tumor microenvironment (TME) represents a complex paradox of both immunosuppressed state with immune evasion tactics via Treg and M2 macrophages (7, 8), while clinical studies also report immune active state with active T cell infiltration and antigen presentation (9). The presence of tumor-infiltrating lymphocytes (TIL) in the tumor microenvironment correlated with improved five-year survival in epithelial ovarian cancer (9). Moreover, higher levels of immune effector cells in cancer tissues, including CD8+ T cells, Natural Killer (NK) cells, and Vγ9Vδ2T-cells, were associated with favorable clinical outcomes for EOC patients (10, 11). A positive clinical outcome is determined by the balance between immune activation and the inflammatory tumor microenvironment. As the latter favors the development of immune suppression, reducing the maturation of myeloid cells, inducing the emergence of regulatory cells, and thus reducing the effector function of lymphocytes that leads to immune evasion and cancer progression (12). Although chemotherapeutic treatment helps in reducing the disease burden, it also affects the immune system (13). It is also evident that apoptotic cancer cell death induced by chemotherapeutic agents can be immunogenic under certain circumstances, and induced immunogenicity is correlated with the different therapeutic interventions (14, 15). Chemotherapy, paclitaxel, and carboplatin were found to reduce the immunosuppressive cells and enhance the level of IFN-γ in the peritoneal cavity of preclinical mouse models (16, 17). Furthermore, the immunomodulatory effect of chemotherapy was also observed in HGSOC patients with evidence of patient-specific immune activation (18). Neoadjuvant chemotherapy may reduce the tumor-associated immunosuppression by reducing the tumor burden and enhancing the antigen processing and presentation (19). Despite these studies, the effect of standard chemotherapy on NK cell receptors -ligands in HGSOC patients remains poorly characterized. Hence, the present study evaluates the effect of chemotherapy on the expression of functional markers on NK, NKT-like, and T cells, their surface, and soluble ligands in high-grade serous epithelial ovarian cancer (HGSOC) patients.

## 2 Materials and methods

### 2.1 Participant enrollment

The study included HGSOC patients from chemo naïve cohort who underwent primary debulking surgery (PDS), or chemo treated cohort, underwent interval debulking surgery (IDS) between 2017 and 2021 at Saifee Hospital and P.D. Hinduja Hospital & Medical Research Center, Mumbai, India. Every enrolled patient signed the informed consent form, which was approved by the institutional ethics committee of the ICMR-National Institute for Research in Reproductive and Child Health, Mumbai, as well as the ethics committees of both hospitals. Blood and tissue samples were collected in an EDTA vacutainer and RPMI medium, respectively. For comparative analysis, blood samples from age and gender-matched healthy controls (HC) were also collected. Patients with immunological disorders, other cancers, along with ovarian cancer, were excluded from the study (**Supplementary Table 1**).

### 2.2 Single-cell suspension of tissue specimens

Tissue specimens were washed twice with PBS and minced with sterile surgical blades. Enzymatic digestion of tissue specimens was carried out by using 4 ml of collagenase and incubated at 37°C in a water bath for 15 minutes. To pellet the cells, samples were centrifuged at 3000 rpm for 10 minutes. Trypsin EDTA (0.05%) + PBS (4ml) was added to the pellet and incubated at 37°C for 10 minutes. After adding 10%FBS+RPMI, the sample tube was centrifuged at 3000 rpm for 10 minutes. RPMI was used to suspend the pellet, which was then strained through a 40 μm cell strainer. The immunological phenotyping of receptors and their related ligands was carried out using the single-cell suspension.

### 2.3 Immune staining of blood, tissue specimens, and flow cytometry

**Figure 1:**
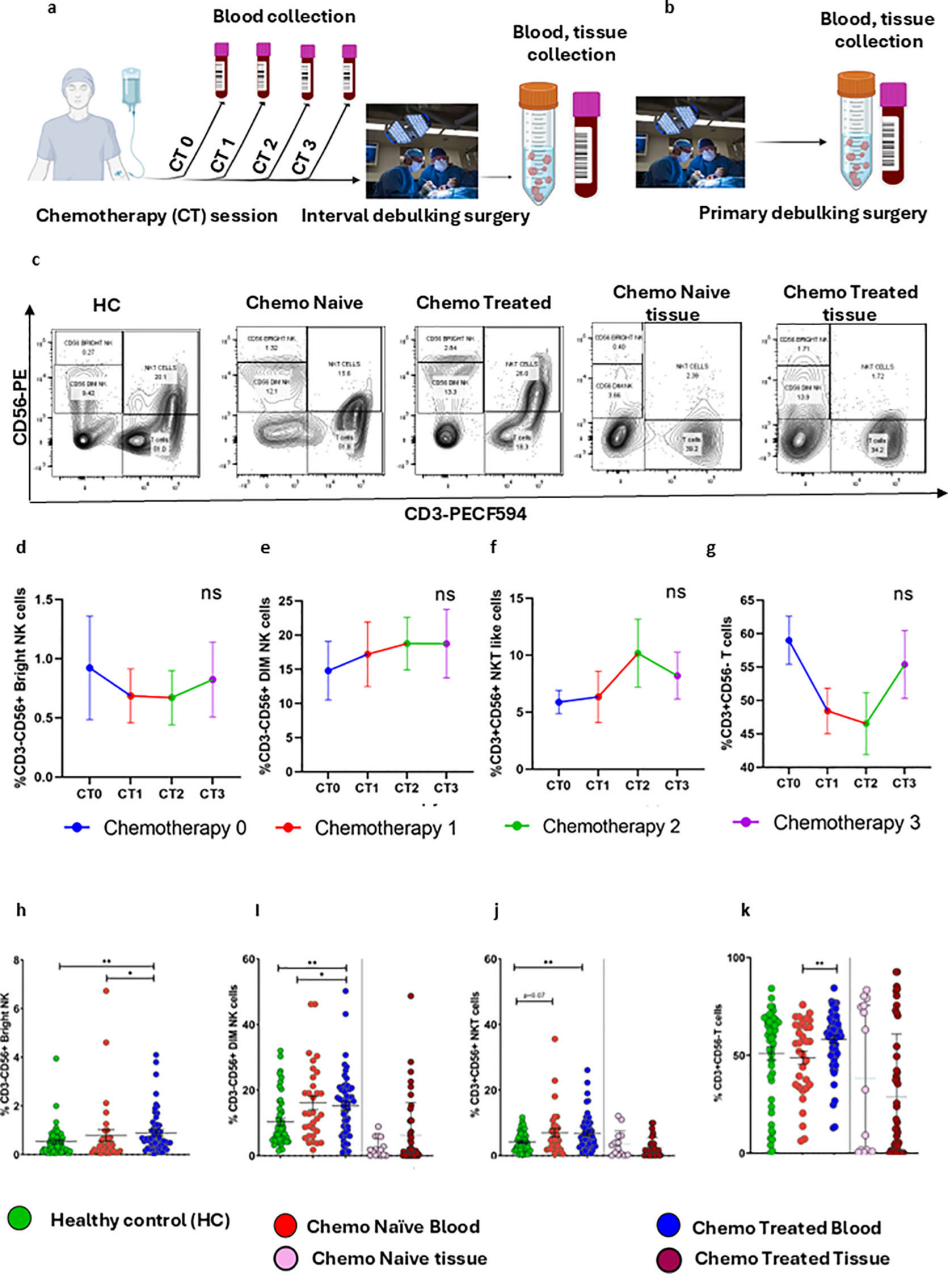
Frequency of immune cells in follow up patients group, chemotherapy-Naïve and chemotherapy-treated HGSOC patients. **(A, B)** Sample collection schedule for the interval and primary debulking surgery group of patients. **(C)** Flow cytometry plots for gating strategies for CD3-CD56+^Bright^ NK, CD3-CD56+^Dim^ NK, NKT-like, and T cells. **(D–G)** Frequencies of CD3-CD56+^Bright^NK cells, CD3-CD56+^Dim^NK cells, CD3+CD56+NKT-like cells, CD3+CD56-T cells in peripheral blood of HGSOC patients during chemotherapy cycles. The repeated Measured ANOVA was used to determine statistical difference between the groups. **(H–K)** Frequencies of CD3-CD56+^Bright^NK cells, CD3-CD56+^Dim^NK cells, CD3+CD56+NKT-like cells, CD3+CD56-T cells in peripheral blood of healthy control, chemo naïve surgical cohort blood, chemo treated surgical cohort blood, chemo untreated surgical cohort tissue, and chemo treated surgical cohort tissue. To determine statistical differences between the groups Mann-Whitney U test was done, and repeated-measure ANOVA was used in follow-up data sets *p<0.05; **p<0.01.

Fluorescent antibody staining was carried out on 150 μL of whole blood and tissue suspension (**Supplementary Table 2**) for 30 minutes at 4°C in the dark. Red blood cells (RBC) were lysed using FACS lysis buffer (BD Biosciences, San Jose, USA) for 15 minutes with intermittent vortexing, and samples were washed twice with staining buffer (0.02% FBS in PBS). Except for RBC lysis, a similar protocol was used to stain tumor-infiltrated immune cells and their cognate ligands. Samples were promptly acquired after staining using the BD FACS Aria™ Fusion flow cytometer (BD Biosciences, San Jose, USA). The data was analyzed using FlowJo software version 10.1. The threshold for positive staining was determined using unstained or fluorescence minus one (FMO) control. A Fixable Violet Dead Cell Stain kit (Invitrogen, Vienna, Austria) was used to remove the dead cells. Samples were analyzed using a sequential gating strategy by using CD45, CD3, CD56 to identify the lymphocytes (**Figure 1A**). For ligand panels, after the exclusion of dead cells, a singlet gate was used to remove the debris and doublets followed by gating for EpCAM+cells to study surface ligands (**Supplementary Figure 1****).**

### 2.4 Procartaplex multiplex immunoassay and ELISA

Cytokines (IL-2, IL-5, IL-6, IL-8, IL-10, 1L-15, IL-27, IFN-γ, TNF-α, PVR, and B7H6) were measured using the Procartaplex Multiplex Immunoassay according to the manufacturer’s protocol (Invitrogen, Vienna, Austria). Briefly, in a 96-well plate, cytokine-specific antibody-coated beads were combined with serum samples and standards. An ELISA plate magnet holder was used throughout the experiment for washing. After washing, enzyme-linked secondary antibodies were added, followed by the addition of streptavidin-R-phycoerythrin (SAPE) to capture the complexes. The plate was washed again, and the cytokine concentration was analyzed using the Liuminex™ instrument (Thermo Fisher Scientific, USA) with a standard curve for quantification.

Serum level of MICA, MICB, and ULBP-1 was analyzed by ELISA according to the manufacturer’s recommendations. (Invitrogen, Vienna, Austria). The absorbance was measured at 450 nm as principle wavelength, by using spectrophotometer. The OD of each sample was used to determine the concentration of these ligands, and standards of known concentrations that were included in the kit were used to plot the standard curve.

### 2.5 Statistical analysis

GraphPad Prism 9.0 (GraphPad Software, San Diego, CA, USA) was used for statistical analysis. Datasets were compared using the Mann-Whitney U-test. Repeated measure ANOVA was used to analyze data generated during the follow-up of patients. Data is reported as mean ± standard error of the mean (SEM). Spearman correlation analysis was carried out to determine the correlation between different variables. To assess the survival difference based on clinical and immunological parameters, we have divided patients based on the median value of immune parameters into “high” and “low” groups. The Kaplan-Meier survival analysis with the log-rank test was performed. Univariate survival analysis (hazard ratio: HR; 95% confidence interval: 95% CI) was performed with the Cox proportional hazards model. The p-values<0.05 were statistically significant.

## 3 Results

### 3.1 Detailed clinicopathological features of HGSOC patients

Ninety HGSOC patients were enrolled in the study. Among them, 33 patients were in chemo naïve surgical cohort and 57 patients were chemo treated surgical cohort. Chemo Naïve surgical cohort patients underwent surgery without prior treatment, while chemotherapy treated surgical patients received three cycles of chemotherapy (Paclitaxel and Carboplatin) before surgery, allowing us to study the immunomodulatory effect of chemotherapy. Seventeen patients in chemotherapy treated surgical cohort were followed during their chemotherapy cycle. To define the disease baseline for chemo Naive group, data from 17 patients at T0 cycle chemotherapy was also included, along with chemo naïve patients data from chemo naïve surgical cohort. The median age was 51.5 (range 34-74) years for chemo naïve surgical cohort and 56 years (32–78 years) for chemo treated surgical cohort (**Supplementary Table 1**-Detailed clinical characteristics of the enrolled patients).

Eighty patients (88.8%) were diagnosed in the advanced stage of the disease, stage (III & IV). The dissemination of tumor cells was observed in the ascitic fluid of 44 patients (48.8%). Lymph node metastases were found in 41 individuals, which accounts for 45.5% of all enrolled HGSOC patients. Their performance status as per the ECOG scale ranged from 0 to 2. Fifty age-matched healthy (Median age: 51, Range: 30–64 years) volunteers were also enrolled as controls.

### 3.2 Lymphocyte distribution in follow-up group, chemo naïve and treated cohorts of HGSOC patients

The sample collection schedule (**Figures 1A, B**) outlines for immune phenotyping performed during chemotherapy sessions, chemo treated and chemo naïve surgical cohort, allowing us to understand longitudinal immunomodulatory effects of chemotherapy during chemotherapy cycles in peripheral blood and further validation in cross sectional surgical cohort on both circulating and tumor-infiltrating immune cells, as well as soluble parameters. Representative flow cytometry plots depict the gating strategy in peripheral blood and tumor samples from different patient groups (**Figure 1C**).

During chemotherapy session, we did not observe major change in immune cell frequency, except a trend of reduced CD3+T cells CT0 to CT2 while reversal of this effect was seen after CT3 (**Figures 1D–G**). However, chemotherapy associated immune alterations were observed in chemotherapy treated cross sectional patients cohorts. The certain immune subsets, like the CD3-CD56+^Bright^ NK (p = 0.042) cells and CD3+CD56-T (p = 0.039) cells, were increased in the peripheral blood of patients in chemotherapy treated surgical cohort (**Figures 1H, K**). Additionally, other subsets CD3-CD56+^Dim^NK (chemo naïve, p = 0.0415; chemo treated = 0.0023) and CD3+CD56+ NKT (chemo naive, p = 0.0416; chemo treated, p = 0.0031) cells were increased in the peripheral blood of both groups compared to healthy controls (**Figures 1I, J**). However, no immune alteration was seen in tumor-infiltrated immune subsets (**Figures 1I–K**).

#### 3.2.1 Immune receptor dynamics during chemotherapy session on CD3-CD56+^Bright^ and Dim NK cells

**Figure 2:**
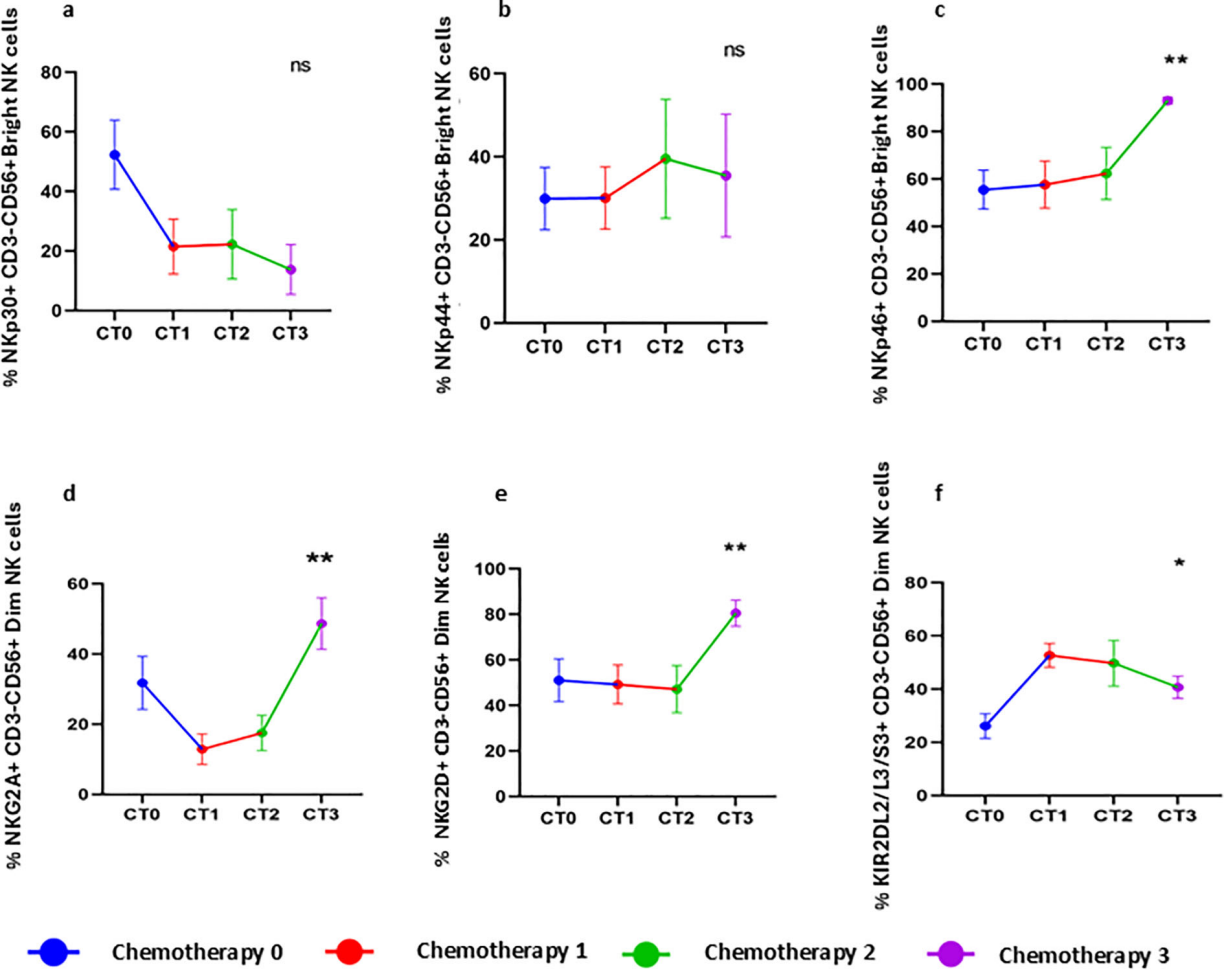
Chemotherapy induced receptor alteration in NK cell subsets follow up of HGSOC patients during chemotherapy sessions. **(A–C)** Expression of NKp30, NKp44, and NKp46 on CD3-CD56+^Bright^ NK cells during chemotherapy cycles. **(D–F)** Expression of NKG2A, NKG2D, and KIR2DL2/L3/S3 on CD3-CD56+ Dim NK cells during chemotherapy cycles. To determine statistical differences between the groups Mann-Whitney U test was done, and repeated-measure ANOVA was used in follow-up data sets *p<0.05; **p<0.01.

To investigate chemotherapy mediated immune alteration, patients were followed during chemotherapy cycles to understand the immune modulatory effect and to determine the specific time point where immune alterations occurred. Notably, NKp46+CD3-CD56+^Bright^ (p =0.0069) cells were increased after the third chemotherapy cycles, while other receptors remained mostly unchanged on CD3-CD56+^Bright^ NK cells (**Figures 2A–C**). Additionally, chemotherapy treated group showed significant increase in the frequency of NKG2A+CD3-CD56+^Dim^NK cells (p =0.0068) (**Figure 2D**), NKG2D+CD3-CD56+^Dim^NKcells (p =0.002) (**Figure 2E**), and KIR2DL2/L3+CD3-CD56+^Dim^NK cells (p =0.0444) (**Figure 2F**), suggesting a more pronounced alteration of the immune profile after the third chemotherapy cycle.

### 3.3 Improved expression of NKp30, NKp46, and KIR2DL2/L3/S3 on CD3-CD56+^Bright^ NK cells in the peripheral blood of chemotherapy-treated surgical cohort

**Figure 3:**
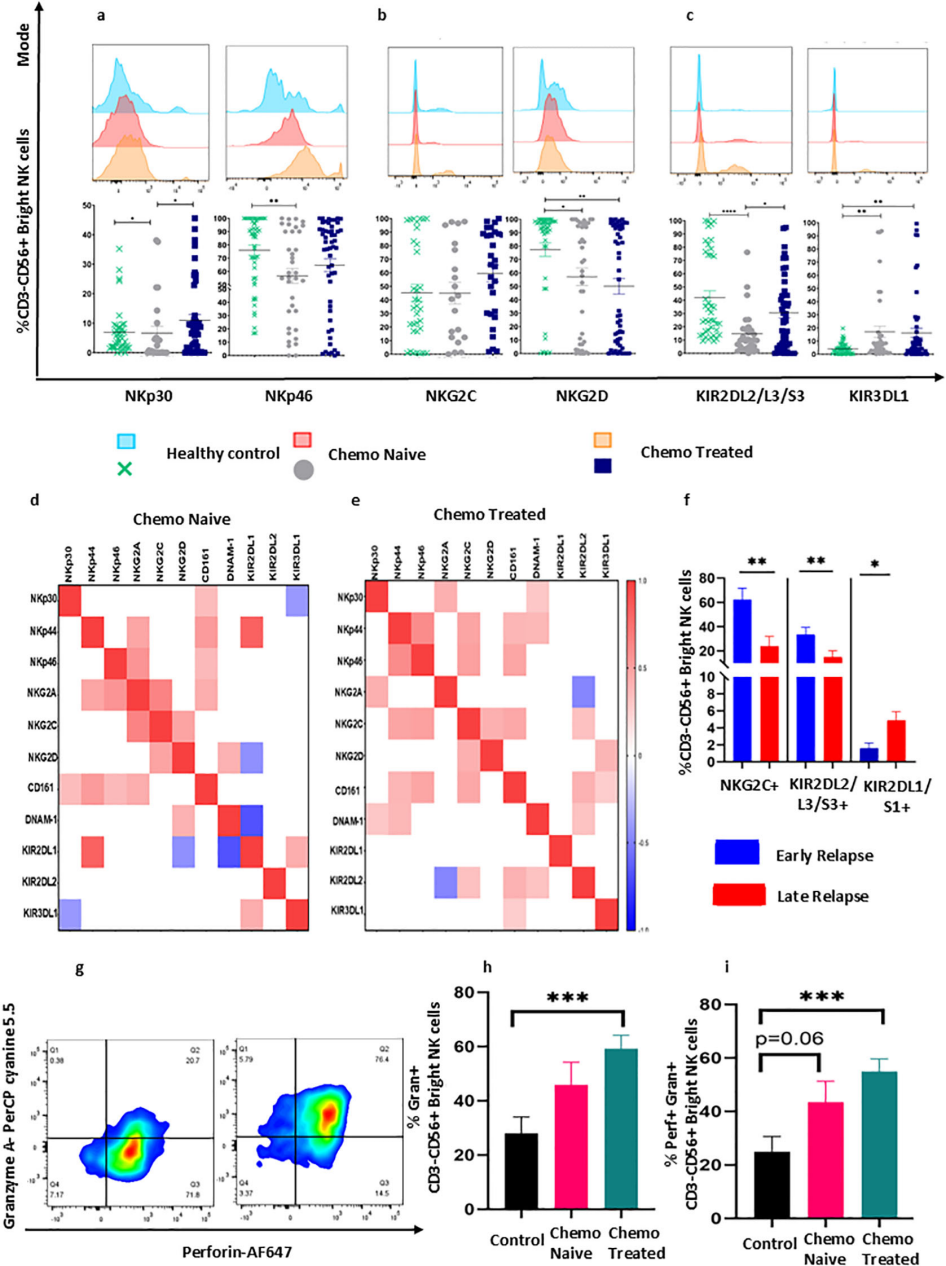
Chemotherapy-induced alteration in CD3-CD56+^Bright^NK cell receptors in peripheral blood of HGSOC patients. **(A)** Expression of NKp30, NKp46, **(B)** NKG2 group of receptors, **(C)** KIR group of receptors in chemo naïve and treated surgical cohort. **(D, E)** Spearman correlation between the receptor expression chemo naïve and chemo treated cohort. **(F)** Frequencies of circulatory NKG2C+CD3-CD56+^Bright^ NK, KIR2DL2/L3/S3+CD3-CD56+^Bright^ NK, and KIR2DL1/S1+CD3-CD56+^Bright^ NK cells in early and late relapse group. **(G)** Representative flow cytometry plots for gating of perforin and granzyme on CD3-CD56+^Bright^ NK cells. **(H, I)** Intracellular granzyme and perforin expression **(H)** Frequency of granzyme+CD3-CD56+^Bright^ NK cell, **(I)** Frequency of dual perforin+granzyme+ CD3-CD56+^Bright^ NK cell in chemo naïve and treated surgical cohort. To determine statistical differences between the groups Mann-Whitney U test was done, and repeated-measure ANOVA was used in follow-up data sets *p<0.05;**p<0.01; ***p<0.001.

Phenotypic characterization of CD3-CD56+^Bright^ NK cells in cross sectional group was conducted to understand and validate the chemotherapy-induced changes in the receptor expression within the immature subset of NK cells. Among NCR receptor group, NKp30+CD3-CD56+^Bright^ , NKp46+CD3-CD56+^Bright^ cells were restored to normal healthy donor level in chemotherapy treated surgical cohort while these subsets NKp30+CD3-CD56+^Bright^ (p = 0.0293), NKp46+CD3-CD56+^Bright^ (p = 0.0047) cells remained significantly reduced in chemotherapy naïve surgical cohort (**Figure 3A**). Additionally, among NKG2 group of receptors, a trend of increased NKG2C+CD3-CD56+^Bright^ cells was observed in chemotherapy treated cohort, whereas KIR2DL2/L3/S3+CD3-CD56+^Bright^ NK cells significantly increased in chemotherapy treated surgical cohort than chemotherapy naïve surgical cohort (**Figure 3C**). Other immune subsets, including NKG2D+CD3-CD56+^Bright^ ,and KIR3DL1+CD3-CD56+^Bright^ cells, did not exhibit chemotherapy-specific changes and were significantly reduced in both chemotherapy-treated and naïve surgical cohorts (**Figures 3B, C**).

Spearman correlation analysis revealed a strong positive correlation between NKG2C and NKp44, NKp46, NKG2D, CD161 and KIR2DL2/L3S3 receptors in the chemotherapy-treated surgical cohort (**Figure 3E**). Adhesion receptor DNAM-1, also showed a strong positive correlation with NKp30, NKp44 and KIR2DL2/L3/S3 in chemotherapy treated surgical cohort (**Figure 3E**). Moreover, KIR2DL2/L3/S3 which had no significant correlation with any of the receptors in chemo naïve surgical cohort, demonstrated significant positive correlation with NKG2C, CD161, and DNAM-1 expression while negative correlation with NKG2A (**Figures 3D, E**). Furthermore, patients were categorized into early and late relapse groups based on median progression-free survival. The percentage of KIR2DL2/L3/S3+CD3-CD56+^Bright^ (p =0.0042) and NKG2C+CD3-CD56+^Bright^ (p =0.0049) cells increased in the early relapse group whereas KIR2DL1/S1+CD3-CD56+^Bright^ (p =0.0224) cells were increased in the late relapse group (**Figure 3F**). Spontaneous cytotoxic potential of NK cells was enhanced with increased level of granzyme+CD3-CD56+^Bright^ NK (p=0.002) and dual Perorin+Granzyme+CD3-CD56+^Bright^ NK cells in chemotherapy treated surgical cohort compared to healthy control (p = 0.004 **Figures 3G–I**).

### 3.4 Restoration of NCR group of receptors and KIR2DL2/L3/S3 on circulatory CD3-CD56+^Dim^NK cells in chemotherapy treated surgical cohort

**Figure 4:**
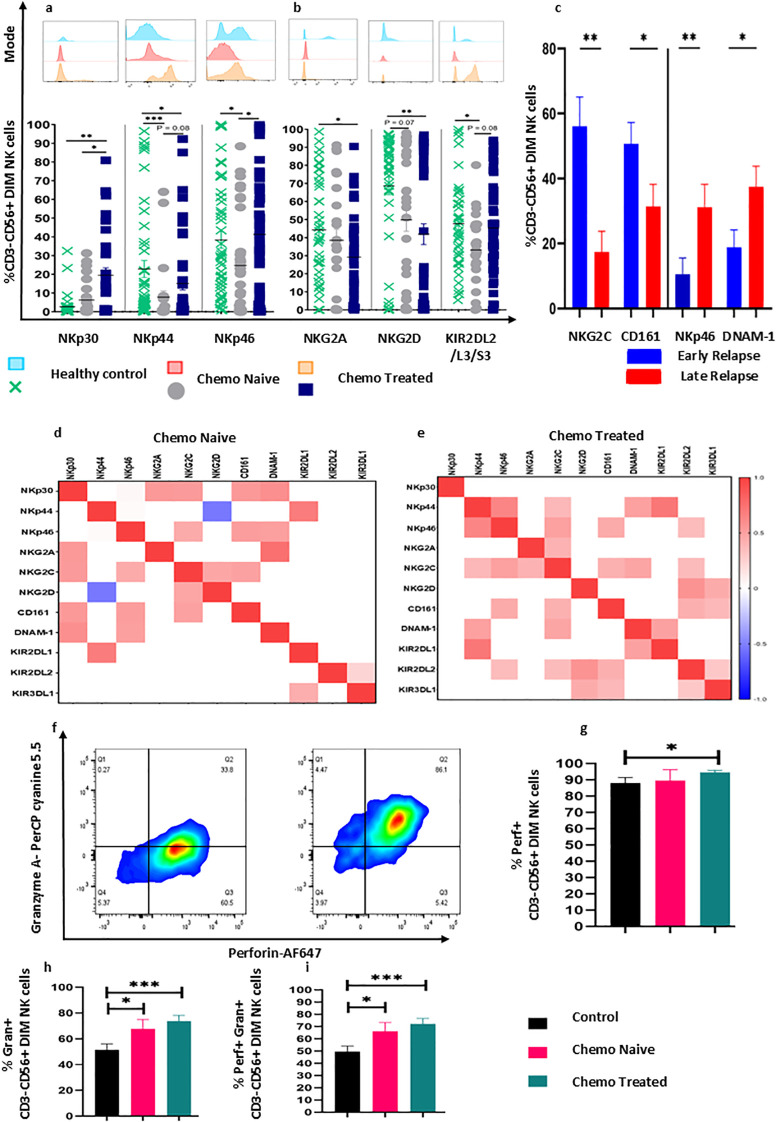
Chemotherapy-induced changes in receptor expression on CD3-CD56+^Dim^NK cells in HGSOC patients. **(A)** Chemotherapy-induced alteration of NCR group of receptors in the peripheral blood of chemo treated cohort. **(B)** Expression of NKG2 group and KIR2DL2/L3/S3 receptors in chemo naïve and treated surgical cohort. **(C)** Increased frequency of circulatory NKG2C+CD3-CD56+^Dim^NK, CD161+CD3-CD56+^Dim^NK cells in early relapse, and tumor infiltrated NKp46+CD3-CD56+^Dim^NK, and DNAM-1+CD3-CD56+^Dim^NK cells in late relapse groups of patients. **(D, E)** Spearman correlation between the receptor expression in chemo naïve and chemo treated cohort. **(F–H)** Representative flow cytometry plots for the gating of perforin and granzyme on CD3-CD56+^Dim^NK cells. **(G)** perforin level on CD3-CD56+^Dim^NK cells, h) Frequency of granzyme+CD3-CD56+^Dim^NK cells, and frequency of perf+granz+CD3-CD56+Dim NK cells in chemotherapy naïve and treated cohort and **(I)** frequency of perf+granz+CD3-CD56+Dim NK cells in chemotherapy naive and treated cohort. To determine statistical difference, the Mann-Whitney U test was used between the independent groups, and repeated-measure ANOVA was used in follow-up data sets *p< 0.05; **p>0.01; ***p<0.001.

Immune profiling of CD3-CD56+^Dim^NK cells revealed complete normalization of most NCR group of receptors to level compared to healthy controls in chemotherapy treated surgical cohort. Moreover, the expression of these receptors (NKp30, p = 0.05, NKp44, p = 0.042, NKp46, p = 0.0211) was also increased in chemotherapy treated surgical cohort compared with chemotherapy naïve cohort (**Figure 4A**) KIR2DL2/L3/S3+CD3-CD56**+**^Dim^NK cells were also comparable to the healthy control levels in chemotherapy treated cohort and showed a trend towards higher expression compared with the chemotherapy naïve surgical cohort (**Figure 4B**), which indicates comprehensible chemotherapy-mediated immune modulation of CD3-CD56**+**^Dim^NK cell in peripheral blood. However, NKG2D+CD3-CD56**+**^Dim^NK cells were reduced (chemo naive, p =0.07; chemo treated, p = 0.0092) in both surgical cohorts (**Figure 4B**). The expression of these receptors was not significantly altered on tumor-infiltrating CD3-CD56+^Dim^NK cells across both patient groups (**Supplementary Figure 2**). In early relapsed patients, we observed an increased frequency of circulatory NKG2C+CD3-CD56+^Dim^NK (p = 0.0079) and CD161+CD3-CD56+^Dim^NK (p =0.0414) cells, whereas tumor-infiltrated NKp46+CD3-CD56+^Dim^NK (p =0.0041) and DNAM-1+CD3-CD56+^Dim^NK (p =0.0287) subsets were elevated in late relapse group (**Figure 4C**). Spearman correlation analysis revealed a strong positive correlation between NKp44, NKp46, NKG2C, NKG2D, CD161 and KIR group of receptors in chemotherapy treated surgical cohort (**Figures 4D, E**). Furthermore, Perf+CD3-CD56+Dim NK cells were increased in chemo treated cohort (p = 0.0215) (**Figure 4G**), whereas Gran+CD3-CD56+Dim NK cells and dual perforin+granzyme+CD3-CD56+^Dim^NK cells increased in both surgical cohorts (**Figures 4G–I**).

### 3.5 Receptor expression profile of CD3+CD56+NKT-like cells in chemo treated and naïve surgical cohort

Unlike NK cell subsets, we did not observe significant alteration of the NCR group of receptors on NKT-like cells. However, within the NKG2 family, NKG2A+CD3+CD56+NKT-like cells showed a trend towards reduction in chemo naïve cohort (**Supplementary Figure 3A**). While, NKG2D+CD3+CD56+NKT-like cells were reduced in both surgical cohorts compared to the control (**Supplementary Figure 3B**). Interestingly, chemotherapy increased the level of CD161+CD3+CD56+NKT-like (p = 0.023) cells in chemo treated surgical cohort relative to chemo naïve surgical cohort (**Supplementary Figure 3C**).

Among KIR receptors, there was an increase in KIR2DL2/L3/S3+CD3+CD56+NKT-like chemo naïve surgical cohort, and KIR3DL1+CD3+CD56+NKT-like cells were increased in both surgical cohorts compared to control (**Supplementary Figures 3D, E**). A Spearman correlation shows a significant negative correlation between NKp44 and NKp46 receptors in the chemo naïve surgical cohort. While, these two NCR group of receptors were positively correlated in chemotherapy treated surgical cohort. Moreover, NKG2C receptor also shows strong positive correlation with NKp44, NKp46, NKG2 group of receptors and DNAM-1 in chemotherapy treated surgical cohort (**Supplementary Figures 3F, G**), suggesting broad receptor alteration post-chemotherapy.

Receptor expression profile of CD3+CD56+NKT-like cells, including CD161, remained relatively stable during chemotherapy cycles (**Supplementary Figure 3H**). Additionally, the percentage of circulatory CD161+CD3+CD56+NKT-like NKp30+CD3+CD56+NKT-like and NKG2C+CD3+CD56+NKT-like cells were high in patients with early relapse, while tumor-infiltrating NKG2A+CD3+CD56+NKT -like cells were higher in late relapsed patients (**Supplementary Figure 3I**).Unlike NK cells, we did not see significant change in perforin and granzyme level in intracellular NKT-like cells (**Supplementary Figures 3J, K**). The expression profile of these receptors was comparable on tumor-infiltrated NKT-like cells between both groups (**Supplementary Figure 4**).

### 3.6 Receptor expression profile of CD3+CD56-T cells in chemo naïve and chemo treated surgical cohort

Receptor expression changes on CD3+CD56-T cells were very similar to NKT-like cells in NKG2 group of receptors. NKG2A+CD3+CD56-T cells were reduced in chemotherapy treated surgical cohort (**Supplementary Figure 5A**) while NKG2D+CD3+CD56-T cells were reduced in both surgical cohorts compared to control (**Supplementary Figure 5B**). Interestingly, chemotherapy selectively reduced KIR3DL1+CD3+CD56-T cells and increased CD161+CD3+CD56- T cells in chemotherapy treated surgical cohort, compared to chemotherapy naïve surgical cohort (**Supplementary Figures 5C, D**). Furthermore, circulatory NKG2C+CD3+CD56-T cells were increased in early-relapsed patients whereas tumor-infiltrated NKG2A+CD3+CD56-T cells, NKp30+CD3+CD56-T cells, and KIR2DL2/L3/S3+CD3+CD56-T cells were increased in late-relapsed patients (**Supplementary Figure 5E**). Although, CD161+CD3+CD56-T cells were increased in cross sectional data of the chemotherapy treated surgical cohort, CD161 expression was not significantly altered during chemotherapy cycles (**Supplementary Figure 5F**). Spearman correlation analysis revealed a strong negative correlation between CD161 and KIR3DL1 in chemotherapy naive surgical cohort (**Supplementary Figure 5G**). Moreover, Adhesion receptor DNAM-1 shows positive correlation with NKG2A, NKG2C and KIR2DL1 in chemotherapy treated surgical cohort (**Supplementary Figure 5H**). Finally, we examined the effect of chemotherapy on granzyme and perforin expression in CD3+CD56-T cells. Co-expression of dual Perforin and Granzyme was increased on CD3+CD56-T cells in both surgical cohorts compared to healthy controls (**Supplementary Figures 5I, J**). The receptor expression was not significantly varied on tumor infiltrated T cells in both surgical cohorts (**Supplementary Figure 6**).

### 3.7 Increased expression of MIC-A on EpCAM+ cells, soluble ligands, and survival of ovarian cancer patients

**Figure 5:**
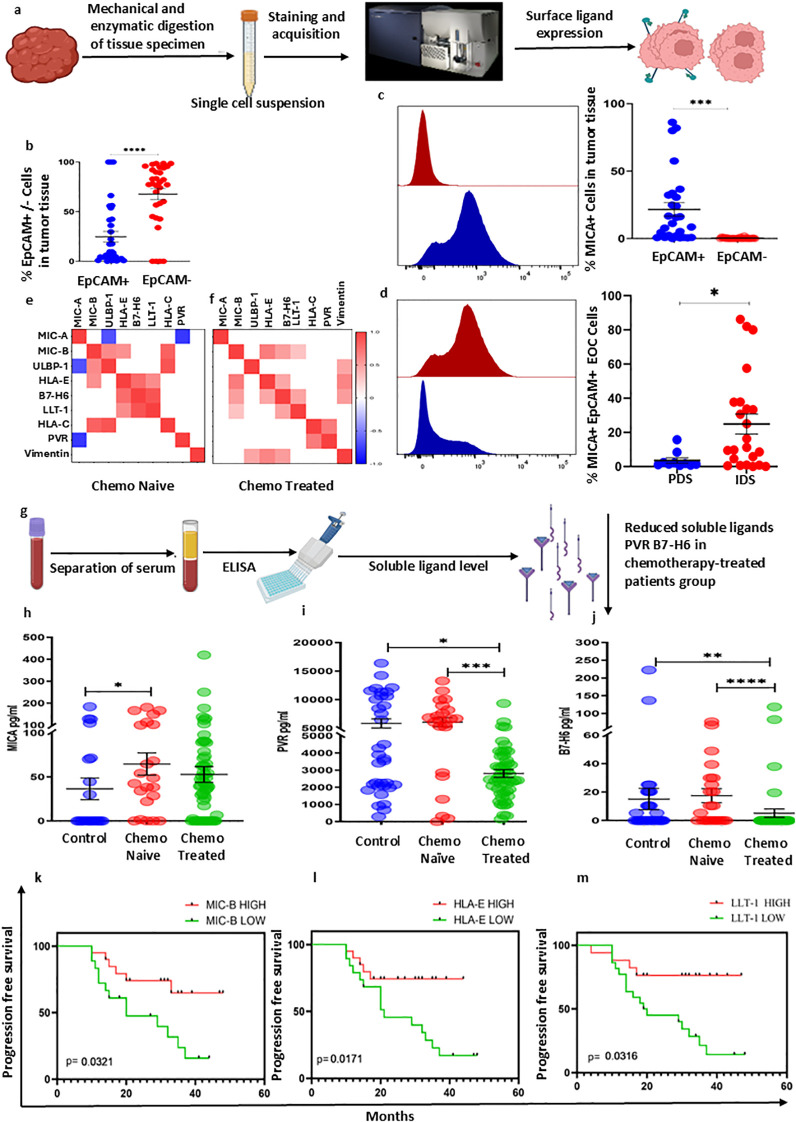
Surface ligand expression on tumor specimens, soluble ligands, and survival. **(A)** Schematic representation of tumor specimens’ collection and processing. **(B)** Frequency of EpCAM^-^ and EpCAM+ cells within tumor specimens in HGSOC patients. **(C, D)** Frequency of MICA+ EpCAM+ cells in chemo naïve and treated cohorts. **(E)** Spearman correlation analysis between the ligand expressions on EpCAM+ cells in chemo naïve cohorts. **(F)** Spearman correlation analysis between ligand expressions on EpCAM+ cells in chemo treated cohorts. **(G)** Schematic representation of the measurement of soluble ligands in serum. **(H)** Serum levels of soluble ligands MICA, **(I)** PVR, and **(J)** LLT-1 in the chemo naïve and treated cohorts. k-m) Progression-free survival of patients stratified by high and low expression of NK cell ligand on tumor cells **(K)** HighMIC-B associated with progression-free survival, **(L)** high HLA-E associated with progression-free survival, and **(M)** high LLT-1 was associated with the progression-free survival of HGSCO patients. To determine statistical difference between the groups, the Mann-Whitney U test was done, Kaplan-Meier survival analysis was done based on ligand expression, *p< 0.05; **p<0.01; ***p<0.001; ****p>0.0001.

We also studied the effect of chemotherapy on surface expression of cognate ligands such as MICA, MICB, ULBP-1, HLA-E, B7-H6, LLT-1, Vimentin, HLA-C, and PVR on EpCAM+ and EpCAM- cells in both groups. A Schematic (**Figure 5A**) shows the processing of tumor specimens to get a single cell suspension and followed by staining and acquisition. We observed a higher proportion of EpCAM- cells in tumor specimen (**Figure 5B**). However, all ligands were significantly upregulated on EpCAM+ cells than EpCAM- cells (**Supplementary Figure 7**). The expression of MICA (p = 0.0012) was increased on EpCAM+ cells (**Figure 5C**). Interestingly, among all ligands, MICA (p = 0.0466)was upregulated on EpCAM+ cells of chemotherapy treated surgical cohort (**Figure 5D**). Spearman correlation analysis revealed significant differences in ligand expression correlations between both surgical cohorts, indicating major shift in expression of these ligands (**Figures 5E, F**).

**Figure 6:**
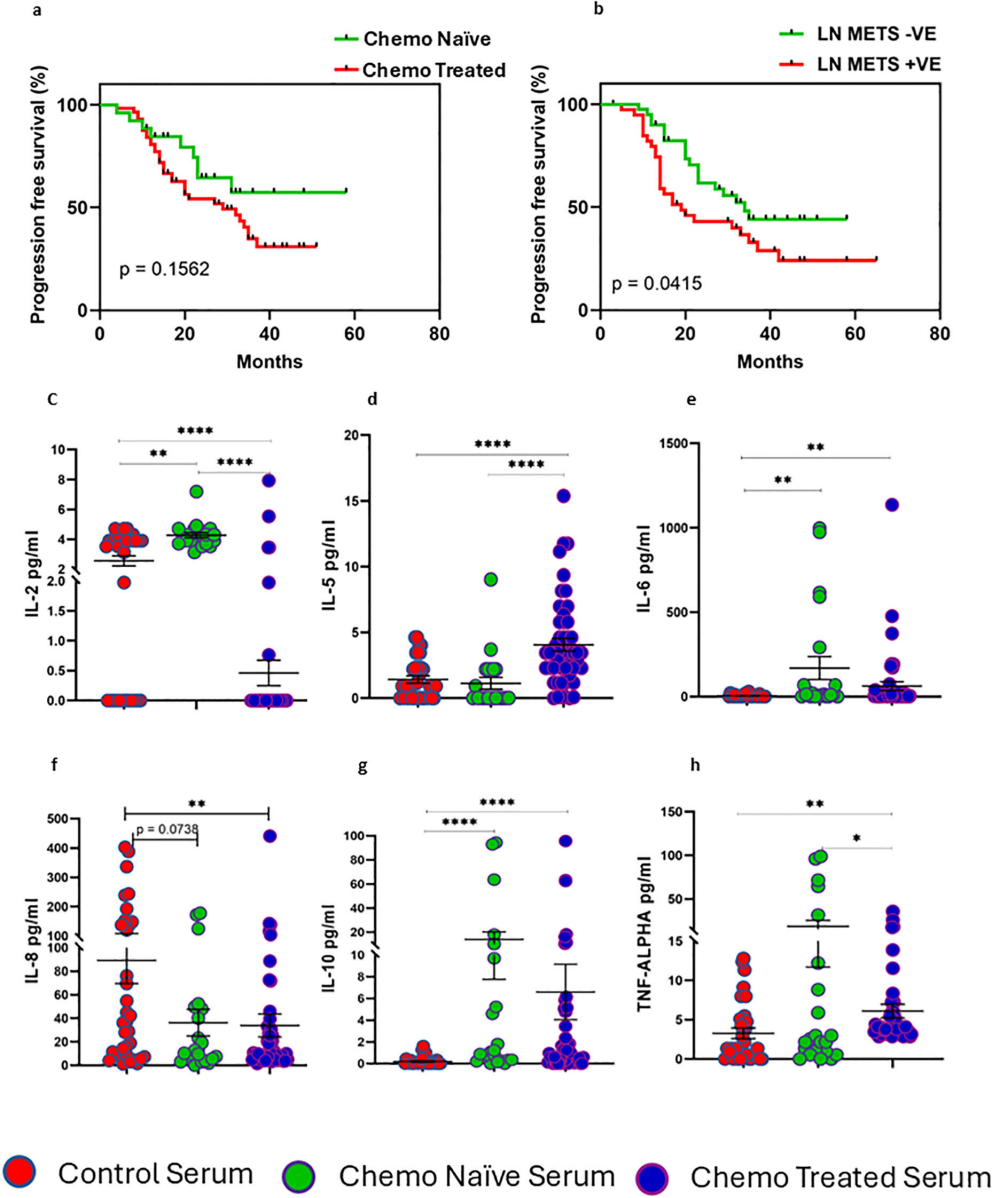
Survival analysis and cytokine level in HGSOC patients. **(A)** Progression-free survival based on treatment mode. **(B)** Poor progression-free survival in patients with lymph node metastasis. **(C–H)** Serum cytokine profile of the chemo naïve and treated surgical cohort. To determine the statistical difference between the groups, Mann-Whitney U test was done, Kaplan Meier survival analysis was done based on treatment mode and lymph node metastasis.*p<0.05; **p<0.01; ***p<0.001; ****p<0.001.

To understand chemotherapy’s impact on the soluble ligands level, we assessed the soluble level of MICA, MICB, ULBP-1, B7-H6, and PVR in serum of both groups, with a schematic shown in (**Figure 5G**). Soluble MICA was elevated in chemo naïve surgical cohort (p = 0.0397) (**Figure 5H**). Interestingly, soluble PVR (p = 0.0002) and B7-H6 (p = <0.0001) were reduced in serum of chemotherapy treated surgical cohort when compared with chemotherapy naïve cohort (**Figures 5I, J**). We hypothesized that chemotherapy-induced MICA upregulation may be associated with the prognosis. To determine this, we have divided patients based on the median expression of ligands into high and low-expressing groups. MICA expression was not significantly associated with progression-free survival [Median; 25 versus 23 months, HR (95% CI) 1.05 (0.325 – 3.136, p = 0.927)]. However, high expression of MICB was associated with better progression-free survival [Median; 20 versus 25 months, HR (95% CI) 3.075(1.234 to 7.663, p = 0.0321) (**Figure 5K**)]. As well as, high expression of LLT-1 was associated with improved progression-free survival [Median 30 versus 21 months, HR (95% CI) 4.106(1.745 to 9.660p = 0.0316), (**Figure 5L**)] and high HLA-E was associated better progression-free survival [Median; 28 versus 21 months HR (95% CI) 3.138(1.306 to 7.540), p = 0.0171) (**Figure 5M**)]. Survival analysis based on clinicopathological characteristics suggests that treatment strategy does not significantly alter the progression free survival of ovarian cancer patients (**Figure 6A**). However, lymph node metastasis was associated with poor progression free survival of HGSOC patients (**Figure 6B**).

### 3.8 Altered serum cytokines in chemotherapy treated and naive surgical cohorts

We analyzed the serum cytokine profile of both groups of HGSOC patients and compared them to those of healthy controls. In chemotherapy-treated surgical cohorts, proinflammatory cytokine IL-2 (p = 0.0001) and TNF-α (p = 0.0442) were reduced (**Figures 6C, H**), while anti-inflammatory cytokine IL-5 (p = 0.0001) was elevated compared to chemotherapy naïve and healthy control (**Figure 6D**). Other cytokines like IL-6, IL-10 were increased in both surgical cohorts compared to healthy control (**Figures 6E–G**). Overall, our findings indicate a reduction in proinflammatory cytokines and an increase in anti-inflammatory cytokines in the serum of the chemotherapy-treated surgical cohort.

## 4 Discussion

This study provides a comprehensive evaluation of the immune alterations induced by chemotherapy in HGSOC patients, longitudinal follow-up of patients during chemotherapy cycles and by comparing the chemotherapy naïve surgical cohort with the chemotherapy-treated surgical cohort of patients. The study reveals how chemotherapy impacts the immune profile, particularly in the peripheral blood and in the tumor microenvironment. Initial investigation during chemotherapy cycles revealed immune modulatory effect on peripheral NK cell subsets, which was further validated on larger cross sectional patients cohort. That chemotherapy exerts a more significant immune modulatory effect on peripheral immune cells than on immune cells in the tumor microenvironment. This differential effect is likely influenced by the tumor heterogeneity, which may obscure the chemotherapy-mediated immune alteration in TME (20). In chemotherapy treated surgical cohort, increased levels of CD3-CD56+^Bright^ NK cells and CD3+CD56-T cells, along with upregulation of receptors like NKG2C, NCRs on NK cells, and CD161+CD3+CD56-T cells, CD161+CD3+CD56+NKT cells, suggest chemotherapy-induced immune activation. This may represent a valuable therapeutic opportunity, where the enhanced function of NK cells and immune reconstitution may give opportunities for further intervention (20, 21). Although, our data demonstrates a notable upregulation in the NCR group of receptors, NKG2C, following chemotherapy in chemotherapy treated surgical cohort. It is important to consider that not all immune parameters exhibit significant change, specifically NCR and KIR group of receptors in chemotherapy treated cohort, indicating chemotherapy-mediated alterations may be very selective (22). This type of differential effect of chemotherapy on immune subsets was reported previously in breast cancer, where B and NK cells were more affected than T cells (23). Certain soluble ligands, like B7-H6 ligands for NKp30, are associated with immune evasion, poor prognosis, and chemoresistance in solid tumors (24). The observed reduced B7H6 levels in chemotherapy-treated cohort potentially aided in restoring NCR receptors and enhancing the NK cell function (25). Studies indicate that targeting these soluble ligands may reprogram NK cells, enhance their response, and potentially boost the efficacy of immune checkpoint therapies (26–29).

These data suggested the direct activation of innate immune response in the chemotherapy treated surgical cohort of HGSOC patients by diminishing inhibitory factors in the circulation. Chemotherapy-induced immune modulation also includes increased expression of dual perforin and granzyme, suggesting enhanced cytolytic potential across NK cell subsets in chemotherapy treated surgical cohort (30). Chemotherapy also upregulated the expression of MICA on EpCAM+ cells. This counters the traditional view of chemotherapy as broadly immunosuppressive, a process that may increase the tumor immunogenicity (31). It was demonstrated that chemotherapy can induce local immune activation, which leads one to believe that chemotherapy can increase the immunogenicity of immune-excluded HGSOC tumors (20). The prognostic significance of immune cells is well-known in various malignancies such as breast and colorectal cancer (32, 33). Immune cells in circulation with receptors like NKp30, NKG2C, and CD161 were prevalent in early relapse cases, whereas tumor-infiltrating immune cells expressing other receptors correlated with late relapse. These findings suggest that the expression and balance of activating and inhibitory receptors influence disease outcome. Further, immune markers may offer prognostic insights that surpass the conventional cancer staging system. Altered NK cell receptor expression was also associated with poor disease outcomes in other malignancies (34, 35). The role of NK cell ligands is well-known in cancer immunosurveillance and immunoediting (36, 37). The findings of our study also indicate the potential prognostic role of MIC-B, HLA-E, and LLT-1 in HGSOC patients. Immune markers were reported as better prognostic indicators than conventional staging systems for solid cancer (38).

Proinflammatory cytokines IL-2 and TNF-α were reduced in chemotherapy-treated patients. Given that high cytokine levels often correlate with poor prognosis (39), their reduction post-chemotherapy may indicate a lowered inflammatory response and a potential decrease in tumor-associated inhibitory factors (40). However, the concurrent expansion of immune cells raises questions about the role of these cytokines in immune cells’ proliferation *in vivo*, suggesting a more complex mechanism at play in ovarian cancer (41).

### 4.1 Limitations of the study

A limitation of the study is the lack of paired pre and post-treatment samples from the same patients in cross sectional cohort may influence the observed differences, specifically in tumor specimens, due to interpatient heterogeneity. The study primarily focuses on chemotherapy’s effect on specific immune markers without exploring the mechanistic pathways behind receptor and ligand restoration. Additionally, it did not detail receptor alterations across the different T cell subsets. Future research should explore chemotherapy’s impact on immune receptors across diverse immune cell subsets in clinical and preclinical models, with an eye towards therapeutic applications.

In conclusion, this study underscores chemotherapy’s capacity to modulate the immune profile in HGSOC patients. These findings may provide valuable details for combination therapies based on chemotherapy-induced immune reactivation and potentially improve the patient’s outcome.

## Data Availability

The original contributions presented in the study are included in the article/**Supplementary Material**. Further inquiries can be directed to the corresponding author.

## Supplementary material

Supplementary Figure 1 **(A)** Representative gating strategy for NK cell receptors. **(B)** Representative gating strategy for ligand panel.

Supplementary Figure 2 Tumour infiltrated CD3-CD56+^Dim^ NK cells phenotype in primary chemo naïve and treated cohorts.

Supplementary Figure 3 Chemotherapy-induced receptor alteration on CD3+CD56+NKT-like cells. **(A)** Expression of NKG2A, **(B)** Frequency of NKG2D, **(C)** Frequency of CD161 in chemo naïve and treated cohort. **(D)** Frequency of KIR2DL2/L3/S3 and, **(E)** Frequency of KIR3DL1 in peripheral blood of both groups. **(F, G)** Spearman correlation between the receptor expression in chemotherapy naïve and treated cohorts **(H)** CD161 expression on CD3+CD56+NKT-like cells during chemotherapy sessions. **(I)** Frequency of NKp30, NKG2C, and CD161 on circulatory CD3+CD56+NKT-like cells in the early relapsed group, and Frequency of NKG2A on tumor-infiltrated CD3+CD56+NKT-like cells in late relapsed cases. **(J)** Representative flow cytometry plots for gating of perforin and granzyme on CD3+CD56+NKT-like cells. **(K, L)** Frequency of perforin+granzyme+ CD3+CD56+NKT-like cells in HGSOC patients. To determine statistical difference Mann-Whitney U test was used between the groups, and repeated-measure ANOVA was used in follow-up data sets *p< 0.05; **p<0.01; ***p<0.001.

Supplementary Figure 4 Tumor-infiltrated CD3+CD56+NKT-like cells phenotype in chemo naive and treated cohort.

Supplementary Figure 5 Chemotherapy-induced receptor alterations on CD3+CD56-T cells. **(A)** Frequency of NKG2A, **(B)** NKG2D and, **(C)** KIR3DL1 on CD3+CD56- T cells in chemo naïve and treated cohort **(D)** Chemotherapy-induced changes in CD161 expression on CD3+CD56-T cells in the peripheral blood of the chemotherapy treated cohort. **(E)** Frequency of NKG2C expression on circulatory CD3+CD56- T cells in early relapsed patients, and frequency of NKp30, NKG2A, KIR2DL2/L3/S3 on tumor in filtered CD3+CD56- T cells in late relapsed patients. **(F)** CD161 expression on CD3+CD56+NKT-like cells during chemotherapy sessions. **(G, H)** Spearman correlation between the receptor expression in the chemo naïve and treated cohorts. **(I)** Representative flow cytometry plots for gating of perforin and granzyme on CD3+CD56- T cells. **(J)** Frequency of frequency of dual perforin+granzyme+ CD3+CD56- T cells in HGSOC patients. To determine statistical differences Mann-Whitney U test was used between the groups, and repeated-measure ANOVA was used in follow-up datasets, *p< 0.05; **p<0.01; ***p<0.001.

Supplementary Figure 6 Tumor-infiltrated CD3+CD56- T cell cells phenotype in primary chemotherapy naïve and treated cohort.

Supplementary Figure 7 Ligand expression profile of EpCAM+ HGSOC tumour cells in primary chemotherapy naïve and treated cohort.

Supplementary Figure 8 Ligands expression profile of EpCAM+ and EpCAM- cells in HGSOC patients *p<0.05; **p<0.01; ***p<0.001; ****p<0.0001.

## References

[1] Siegel RL Kratzer TB Giaquinto AN Sung H Jemal A . Cancer statistics, 2025. CA Cancer J Clin. (2025). 75:10–45. doi: 10.3322/caac.21871, PMID: 39817679 PMC11745215

[2] Ranmale S Kumar P Tongaonkar H Mehta S Maniar V Mania-Pramanik J . Chemotherapy-induced alterations in miRNA expression and their prognostic implications in ovarian cancer. Front Oncol. (2025) 15:1580565. doi: 10.3389/fonc.2025.1580565, PMID: 40919157 PMC12411204

[3] Cortez AJ Tudrej P Kujawa KA Lisowska KM . Advances in ovarian cancer therapy. Cancer ChemotherPharmacol. (2018) 81:17–38. doi: 10.1007/s00280-017-3501-8, PMID: 29249039 PMC5754410

[4] Kumar P Ranmale S Mehta S Tongaonkar H Patel V Singh AK . Immune profile of primary and recurrent epithelial ovarian cancer cases indicates immune suppression, a major cause of progression and relapse of ovarian cancer. J Ovarian Res. (2023) 16:114 doi: 10.1186/s13048-023-01192-4, PMID: 37322531 PMC10268537

[5] Preston CC Goode EL Hartmann LC Kalli KR Knutson KL . Immunity and immune suppression in human ovarian cancer. Immunotherapy. (2011) 3:539–56. doi: 10.2217/imt.11.20, PMID: 21463194 PMC3147144

[6] Bamias A Gavalas NG Karadimou A Dimopoulos MA . Immune response in ovarian cancer: How is the immune system involved in prognosis and therapy: Potential for treatment utilization. Clin Dev Immunol. (2010) 2010:791603 doi: 10.1155/2010/791603, PMID: 21318181 PMC3034919

[7] Pawłowska A Rekowska A Kuryło W Pańczyszyn A Kotarski J Wertel I . Current understanding on why ovarian cancer is resistant to immune checkpoint inhibitors. Int J Mol Sci. (2023) 24:10859 doi: 10.3390/ijms241310859, PMID: 37446039 PMC10341806

[8] Ghoneum A Afify H Salih Z Kelly M Said N . Role of tumor microenvironment in the pathobiology of ovarian cancer: Insights and therapeutic opportunities. Cancer Med. (2018) 7:5047–56. doi: 10.1002/cam4.1741, PMID: 30133163 PMC6198242

[9] Pautu JL Kumar L . Intratumoral T cells and survival in epithelial ovarian cancer. Natl Med J India. (2003) 16:150–1. 12929858

[10] Garzetti GG Cignitti M Ciavattini A Fabris N Romanini C . Natural killer cell activity and progression-free survival in ovarian cancer. GynecolObstet Invest. (1993) 35:118–20. doi: 10.1159/000292678, PMID: 8383627

[11] Thedrez A Lavoué V Dessarthe B Daniel P Henno S Jaffre I . A quantitative deficiency in peripheral blood Vγ9Vδ2 cells is a negative prognostic biomarker in ovarian cancer patients. PloS One. (2013) 8:1–12. doi: 10.1371/journal.pone.0063322, PMID: 23717410 PMC3662688

[12] Lavoué V Thédrez A Levêque J Foucher F Henno S Jauffret V . Immunity of human epithelial ovarian carcinoma: The paradigm of immune suppression in cancer. J Transl Med. (2013) 11:1–12. doi: 10.1186/1479-5876-11-1474, PMID: 23763830 PMC3683338

[13] Zitvogel L Apetoh L Ghiringhelli F Kroemer G . Immunological aspects of cancer chemotherapy. Nat Rev Immunol. (2008) 8:59–73. doi: 10.1038/nri2216, PMID: 18097448

[14] Obeid M Tesniere A Ghiringhelli F Fimia GM Apetoh L Perfettini J-L . Calreticulin exposure dictates the immunogenicity of cancer cell death. Nat Med. (2007) 13:54–61. doi: 10.1038/nm1523, PMID: 17187072

[15] Chaput N De Botton S Obeid M Apetoh L Ghiringhelli F Panaretakis T . Molecular determinants of immunogenic cell death: surface exposure of calreticulin makes the difference. J Mol Med (Berl). (2007) 85:1069–76. doi: 10.1007/s00109-007-0214-1, PMID: 17891368

[16] Vankerckhoven A Baert T Riva M De Bruyn C Thirion G Vandenbrande K . Type of chemotherapy has substantial effects on the immune system in ovarian cancer. Transl Oncol. (2021) 14:101076 doi: 10.1016/j.tranon.2021.101076, PMID: 33770618 PMC8022256

[17] Waidhauser J Schuh A Trepel M Schmälter A-K Rank A . Chemotherapy markedly reduces B cells but not T cells and NK cells in patients with cancer. Cancer Immunol Immunother. (2020) 69:147–57. doi: 10.1007/s00262-019-02449-y, PMID: 31900508 PMC11027838

[18] Vanguri R Benhamida J Young JH Li Y Zivanovic O Chi D . Understanding the impact of chemotherapy on the immune landscape of high-grade serous ovarian cancer. Gynecol Oncol Rep. (2022) 39:100926 doi: 10.1016/j.gore.2022.100926, PMID: 35146104 PMC8801989

[19] Liu M Tayob N Penter L Sellars M Tarren A Chea V . Improved T-cell immunity following neoadjuvant chemotherapy in ovarian cancer. Clin Cancer Res. (2022) 28:3356–66. doi: 10.1158/1078-0432.CCR-21-2834, PMID: 35443043 PMC9357177

[20] Jiménez-Sánchez A Cybulska P Mager KLV Koplev S Cast O Couturier DL . Unraveling tumor–immune heterogeneity in advanced ovarian cancer uncovers immunogenic effect of chemotherapy. Nat Genet. (2020) 52:582–93. doi: 10.1038/s41588-020-0630-5, PMID: 32483290 PMC8353209

[21] Garcia-Iglesias T del Toro-Arreola A Albarran-Somoza B del Toro-Arreola S Sanchez-Hernandez PE Ramirez-Dueñas M . Low NKp30, NKp46 and NKG2D expression and reduced cytotoxic activity on NK cells in cervical cancer and precursor lesions. BMC Cancer. (2009) 9:1–8. doi: 10.1186/1471-2407-9-186, PMID: 19531227 PMC2704222

[22] Kumar P Ranmale S Tongaonkar H Mania-Pramanik J . Immune profile of blood, tissue and peritoneal fluid: A comparative study in high grade serous epithelial ovarian cancer patients at interval debulking surgery. Vaccines (Basel). (2022) 10:2121 doi: 10.3390/vaccines10122121, PMID: 36560531 PMC9784879

[23] Massa C Karn T Denkert C Schneeweiss A Hanusch C Blohmer JU . Differential effect on different immune subsets of neoadjuvant chemotherapy in patients with TNBC. J Immunother Cancer. (2020) 8:e001261 doi: 10.1136/jitc-2020-001261, PMID: 33199511 PMC7670944

[24] Semeraro M Rusakiewicz S Minard-Colin V Delahaye NF Enot D Vély F . Clinical impact of the NKp30/B7-H6 axis in high-risk neuroblastoma patients. Sci Transl Med. (2015) 7:283ra55 doi: 10.1126/scitranslmed.aaa2327, PMID: 25877893

[25] Ha H Bang JH Nam AR Park JE Jin MH Bang YJ . Dynamics of Soluble Programmed Death-Ligand 1 (sPDL1) during Chemotherapy and Its Prognostic Implications in Cancer Patients: Biomarker Development in Immuno-oncology. Cancer Res Treat. (2019) 51:832–40. doi: 10.4143/crt.2018.311. PMID: 30309223 PMC6473273

[26] Chitadze G Bhat J Lettau M Janssen O Kabelitz D . Generation of soluble NKG2D ligands: proteolytic cleavage, exosome secretion and functional implications. Scand J Immunol. (2013) 78:120–9. doi: 10.1111/sji.12072, PMID: 23679194

[27] Basher F Dhar P Wang X Wainwright DA Zhang B Sosman J . Antibody targeting tumor-derived soluble NKG2D ligand sMIC reprograms NK cell homeostatic survival and function and enhances melanoma response to PDL1 blockade therapy. J Hematol Oncol. (2020) 0:1–16. doi: 10.1186/s13045-020-00896-0, PMID: 32517713 PMC7285527

[28] Zhang J Larrocha PS Zhang B Wainwright D Dhar P Wu JD . Antibody targeting tumor-derived soluble NKG2D ligand sMIC provides dual co- stimulation of CD8 T cells and enables sMIC + tumors respond to PD1/PD-L1 blockade therapy. J. immunotherap. cancer. (2019) 7:1–15. doi: 10.1186/s40425-019-0693-y, PMID: 31446896 PMC6709558

[29] Attwood MM Schiöth HB . Soluble ligands as drug targets. Nat Rev Drug Discov. (2020)19:695–710. doi: 10.1038/s41573-020-0078-4, PMID: 32873970

[30] Voskoboinik I Whisstock JC Trapani JA . Perforin and granzymes: Function, dysfunction and human pathology. Nat Rev Immunol. (2015) 15:388–400. doi: 10.1038/nri3839, PMID: 25998963

[31] Cosiski Marana HR Santana da Silva J Moreira de Andrade J . NK cell activity in the presence of IL-12 is a prognostic assay to neoadjuvant chemotherapy in cervical cancer. Gynecol Oncol. (2000) 78:318–23. doi: 10.1006/gyno.2000.5878, PMID: 10985887

[32] Iseki Y Shibutani M Maeda K Nagahara H Tamura T Ohira G . The impact of the preoperative peripheral lymphocyte count and lymphocyte percentage in patients with colorectal cancer. Surg Today. (2017) 47:743–54. doi: 10.1007/s00595-016-1433-2, PMID: 27783149

[33] Yang J Xu J Ying E Sun T . Predictive and prognostic value of circulating blood lymphocyte subsets in metastatic breast cancer. Cancer Med. (2019) 8:492–500. doi: 10.1002/cam4.1891, PMID: 30632318 PMC6382707

[34] Rocca YS Roberti MP Juliá EP Pampena MB Bruno L Rivero S . Phenotypic and functional dysregulated blood NK cells in colorectal cancer patients can be activated by cetuximab plus IL-2 or IL-15. Front Immunol. (2016) 7:413. doi: 10.3389/fimmu.2016.00413, PMID: 27777574 PMC5056190

[35] Fregni G Messaoudene M Fourmentraux-Neves E Mazouz-Dorval S Chanal J Maubec E . Phenotypic and functional characteristics of blood natural killer cells from melanoma patients at different clinical stages. PloS One. (2013) 8:1–9. doi: 10.1371/journal.pone.0076928, PMID: 24204708 PMC3799851

[36] McGilvray RW Eagle RA Rolland P Jafferji I Trowsdale J Durrant LG . ULBP2 and RAET1E NKG2D ligands are independent predictors of poor prognosis in ovarian cancer patients. Int J Cancer. (2010) 127:1412–20. doi: 10.1002/ijc.25156, PMID: 20054857

[37] Madjd Z Spendlove I Moss R Bevin S Pinder SE Watson NFS . Upregulation of MICA on high-grade invasive operable breast carcinoma. Cancer Immun. (2007) 7:17. PMC2935745 17948965

[38] Bindea G Mlecnik B Fridman WH Galon J . The prognostic impact of anti-cancer immune response: a novel classification of cancer patients. Semin Immunopathol. (2011) 33:335–40. doi: 10.1007/s00281-011-0264-x, PMID: 21461991 PMC3139059

[39] Dobrzycka B Mackowiak-Matejczyk B Terlikowska KM Kulesza-Bronczyk B Kinalski M Terlikowski SJ . Serum levels of IL-6, IL-8 and CRP as prognostic factors in epithelial ovarian cancer. Eur Cytokine Netw. (2013) 24:106–13. doi: 10.1684/ecn.2013.0340, PMID: 24197277

[40] Lee HM Lee HJ Chang JE . Inflammatory cytokine: an attractive target for cancer treatment. Biomedicines. (2022) 10. 2116 doi: 10.3390/biomedicines10092116, PMID: 36140220 PMC9495935

[41] Fehniger TA Bluman EM Porter MM Mrózek E Cooper MA VanDeusen JB . Potential mechanisms of human natural killer cell expansion *in vivo* during low-dose IL-2 therapy. J Clin Invest. (2000). 106:117–124. doi: 10.1172/JCI6218, PMID: 10880055 PMC314354

