# Supplementary figures and images for: Chemotherapy driven alterations in NK cell receptors and ligands in high grade serous ovarian cancer
Source: Front Immunol. 2026 Mar 31;17:1765987. doi: 10.3389/fimmu.2026.1765987 (PMC13076314; doi:10.3389/fimmu.2026.1765987)

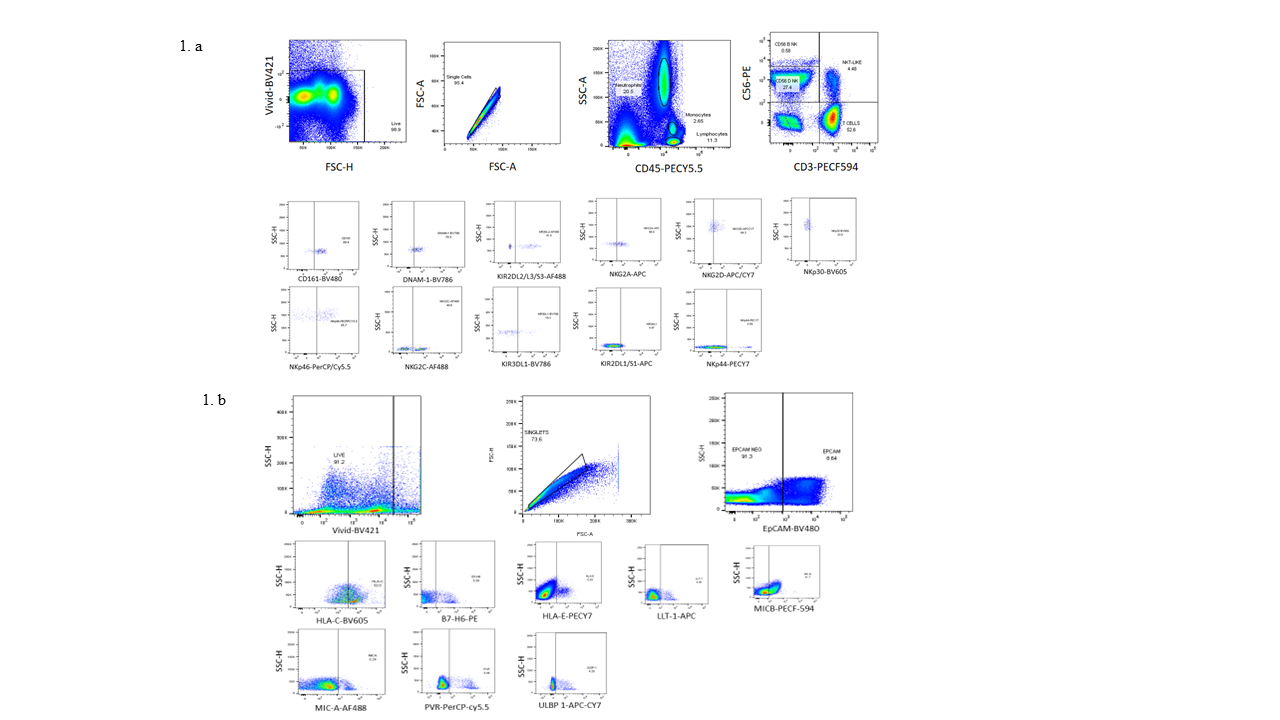

Supplement: Supplementary Figure 1 — (A) Representative gating strategy for NK cell receptors. (B) Representative gating strategy for ligand panel. [file Image1.tif]

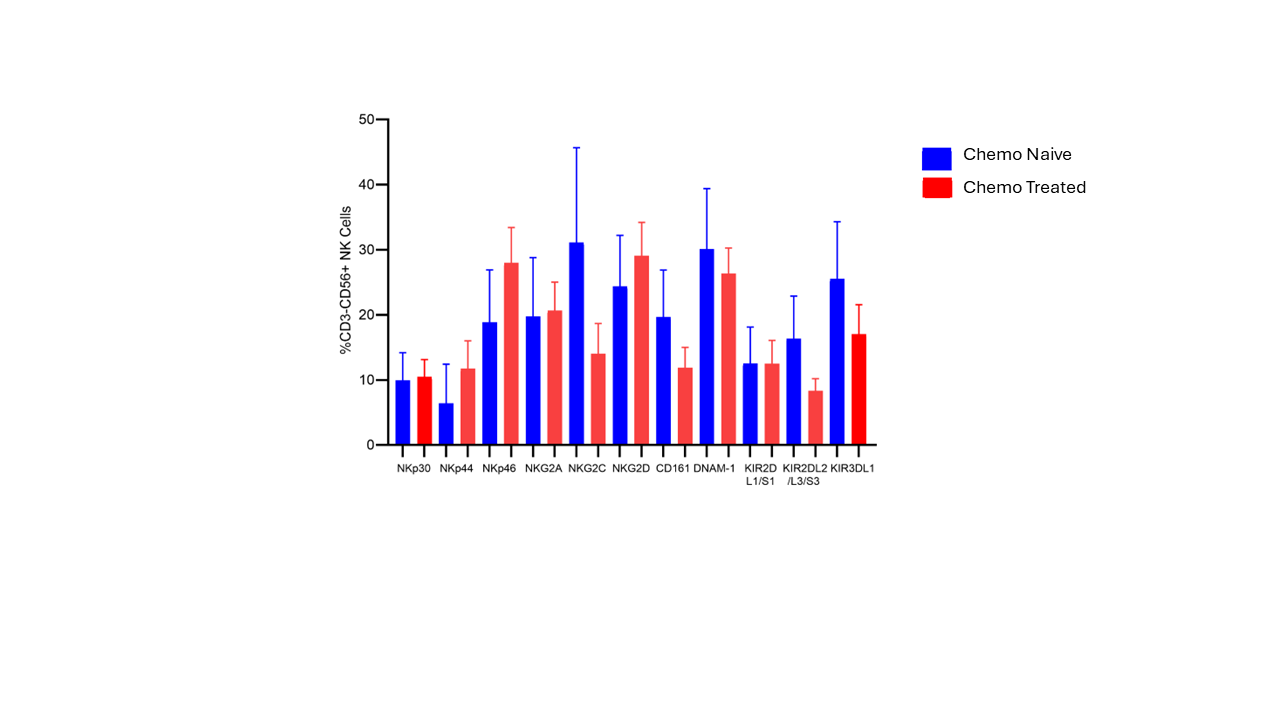

Supplement: Supplementary Figure 2 — Tumour infiltrated CD3-CD56+Dim NK cells phenotype in primary chemo naïve and treated cohorts. [file Image2.tif]

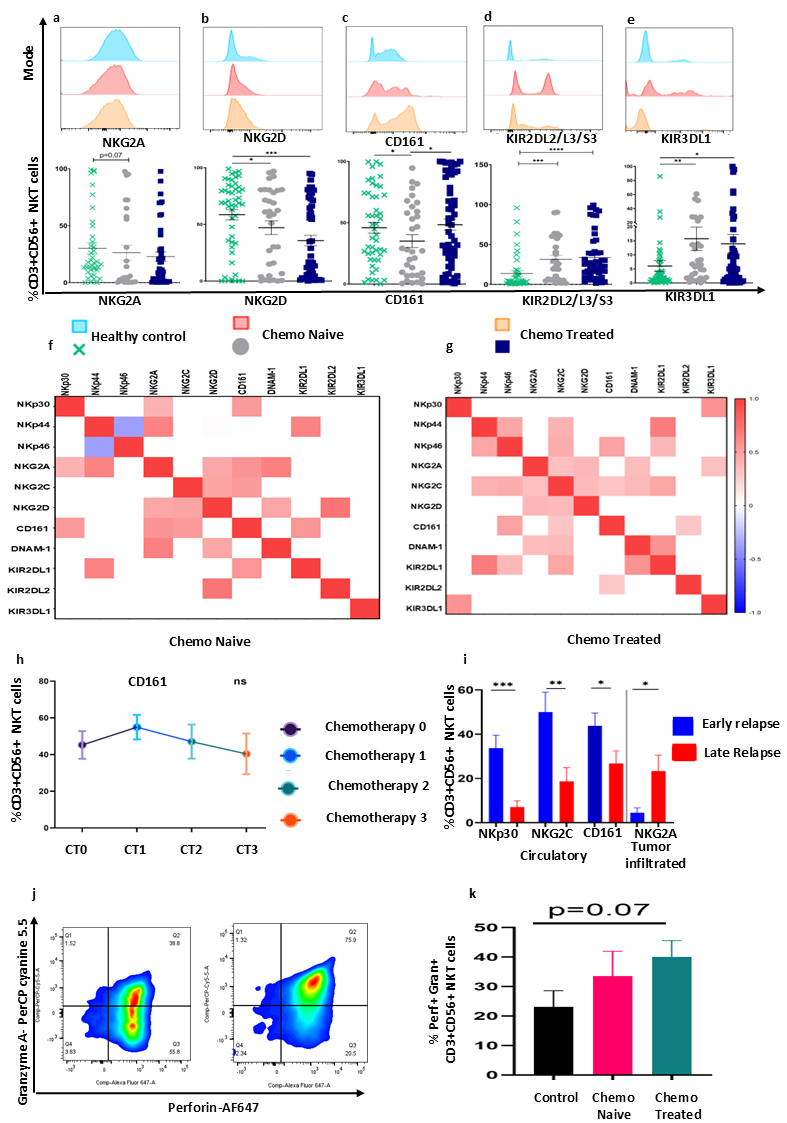

Supplement: Supplementary Figure 3 — Chemotherapy-induced receptor alteration on CD3+CD56+NKT-like cells. (A) Expression of NKG2A, (B) Frequency of NKG2D, (C) Frequency of CD161 in chemo naïve and treated cohort. (D) Frequency of KIR2DL2/L3/S3 and, (E) Frequency of KIR3DL1 in peripheral blood of both groups. (F, G) Spearman correlation between the receptor expression in chemotherapy naïve and treated cohorts (H) CD161 expression on CD3+CD56+NKT-like cells during chemotherapy sessions. (I) Frequency of NKp30, NKG2C, and CD161 on circulatory CD3+CD56+NKT-like cells in the early relapsed group, and Frequency of NKG2A on tumor-infiltrated CD3+CD56+NKT-like cells in late relapsed cases. (J) Representative flow cytometry plots for gating of perforin and granzyme on CD3+CD56+NKT-like cells. (K, L) Frequency of perforin+granzyme+ CD3+CD56+NKT-like cells in HGSOC patients. To determine statistical difference Mann-Whitney U test was used between the groups, and repeated-measure ANOVA was used in follow-up data sets *p< 0.05; **p<0.01; ***p<0.001. [file Image3.tif]

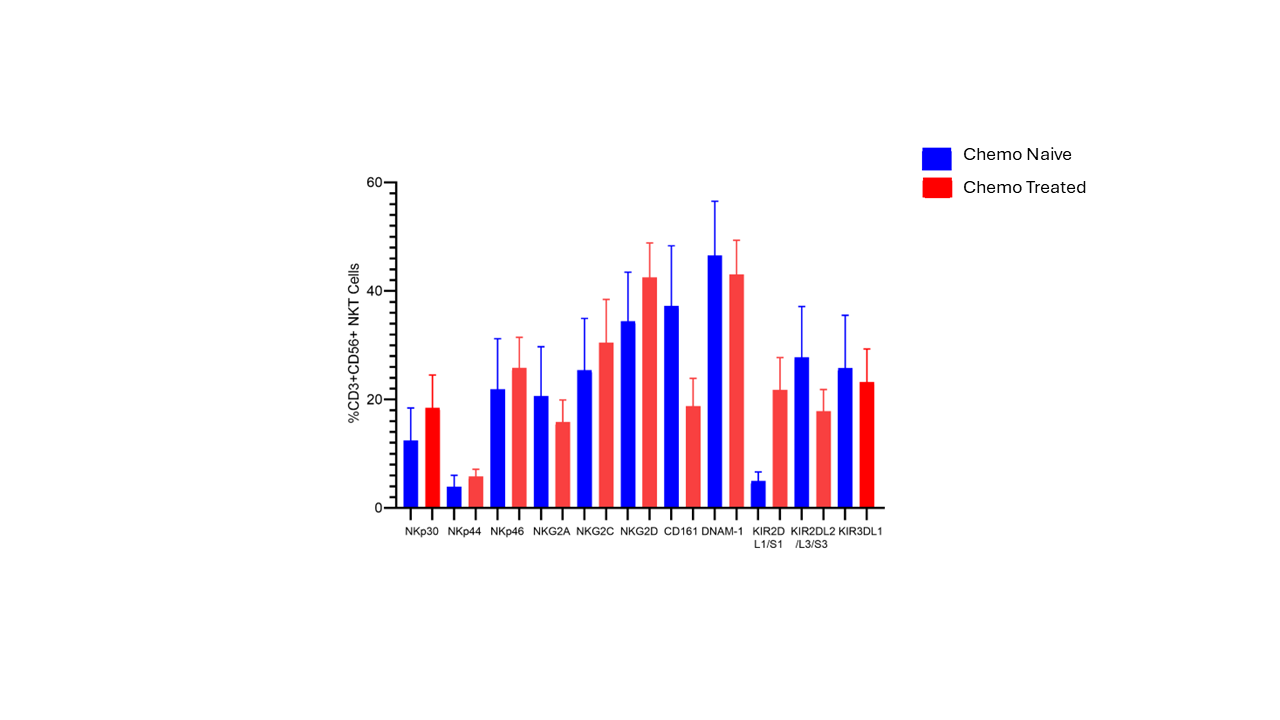

Supplement: Supplementary Figure 4 — Tumor-infiltrated CD3+CD56+NKT-like cells phenotype in chemo naive and treated cohort. [file Image4.tif]

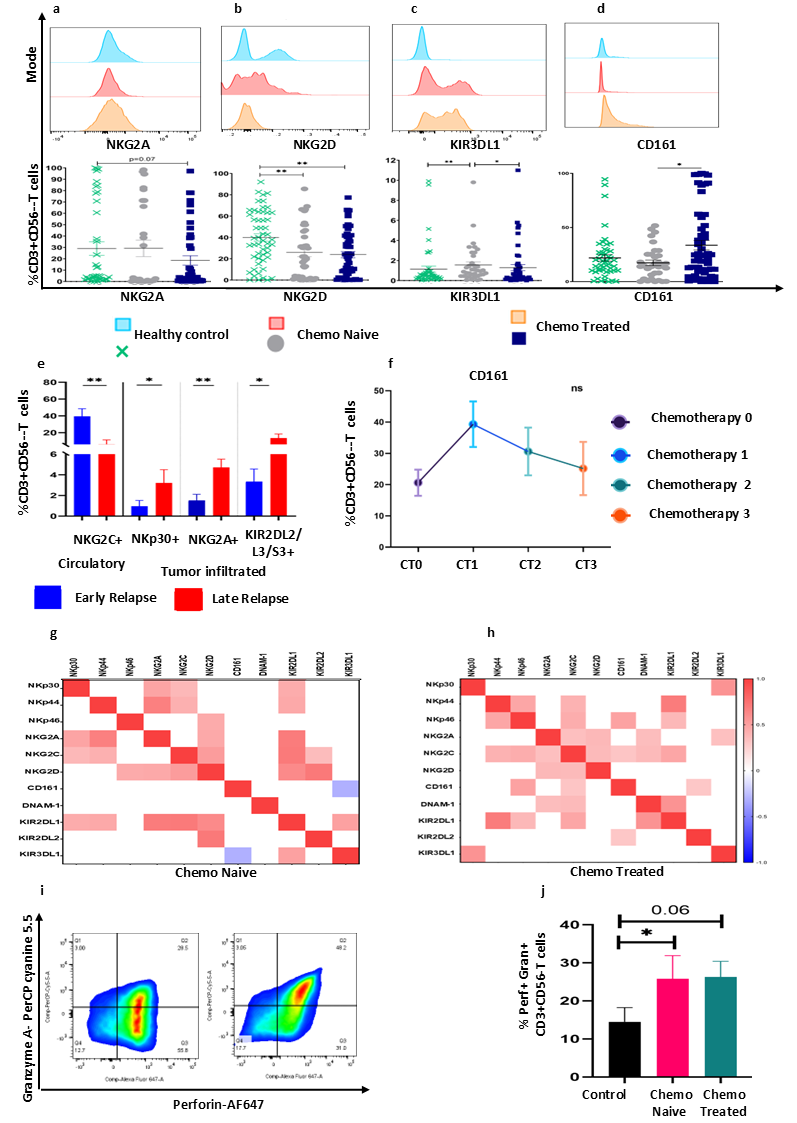

Supplement: Supplementary Figure 5 — Chemotherapy-induced receptor alterations on CD3+CD56-T cells. (A) Frequency of NKG2A, (B) NKG2D and, (C) KIR3DL1 on CD3+CD56- T cells in chemo naïve and treated cohort (D) Chemotherapy-induced changes in CD161 expression on CD3+CD56-T cells in the peripheral blood of the chemotherapy treated cohort. (E) Frequency of NKG2C expression on circulatory CD3+CD56- T cells in early relapsed patients, and frequency of NKp30, NKG2A, KIR2DL2/L3/S3 on tumor in filtered CD3+CD56- T cells in late relapsed patients. (F) CD161 expression on CD3+CD56+NKT-like cells during chemotherapy sessions. (G, H) Spearman correlation between the receptor expression in the chemo naïve and treated cohorts. (I) Representative flow cytometry plots for gating of perforin and granzyme on CD3+CD56- T cells. (J) Frequency of frequency of dual perforin+granzyme+ CD3+CD56- T cells in HGSOC patients. To determine statistical differences Mann-Whitney U test was used between the groups, and repeated-measure ANOVA was used in follow-up datasets, *p< 0.05; **p<0.01; ***p<0.001. [file Image5.tif]

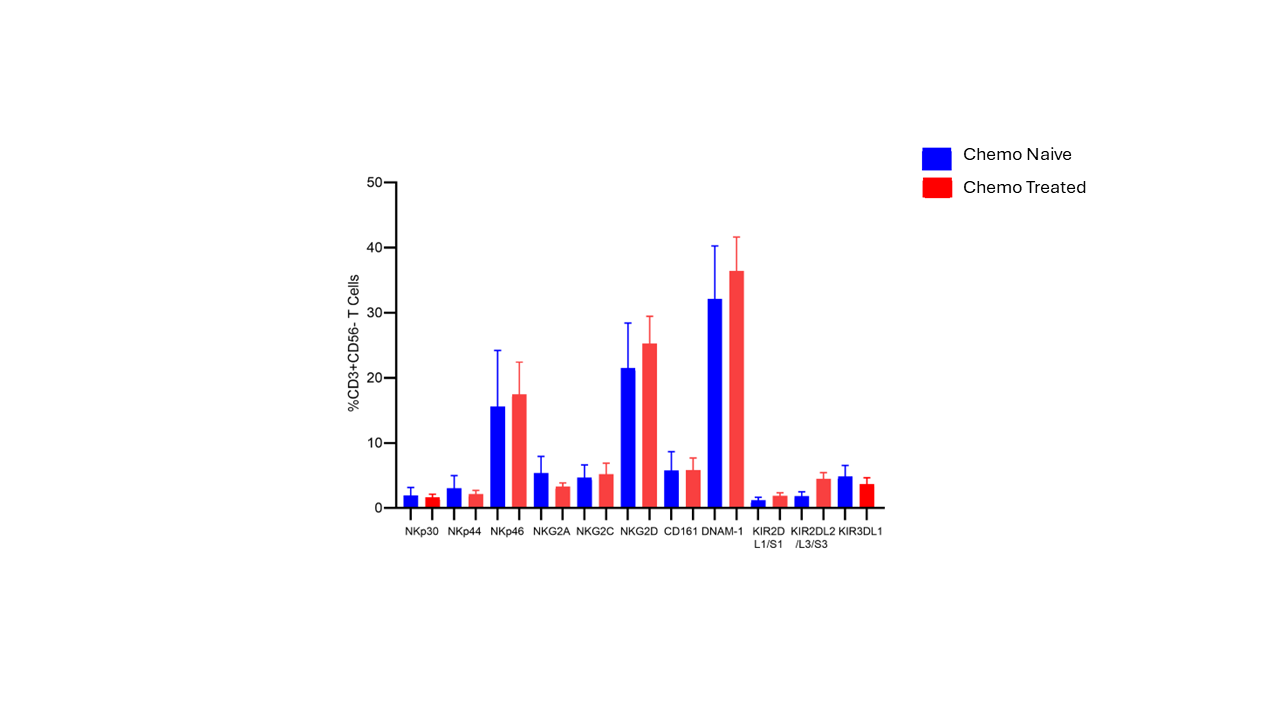

Supplement: Supplementary Figure 6 — Tumor-infiltrated CD3+CD56- T cell cells phenotype in primary chemotherapy naïve and treated cohort. [file Image6.tif]

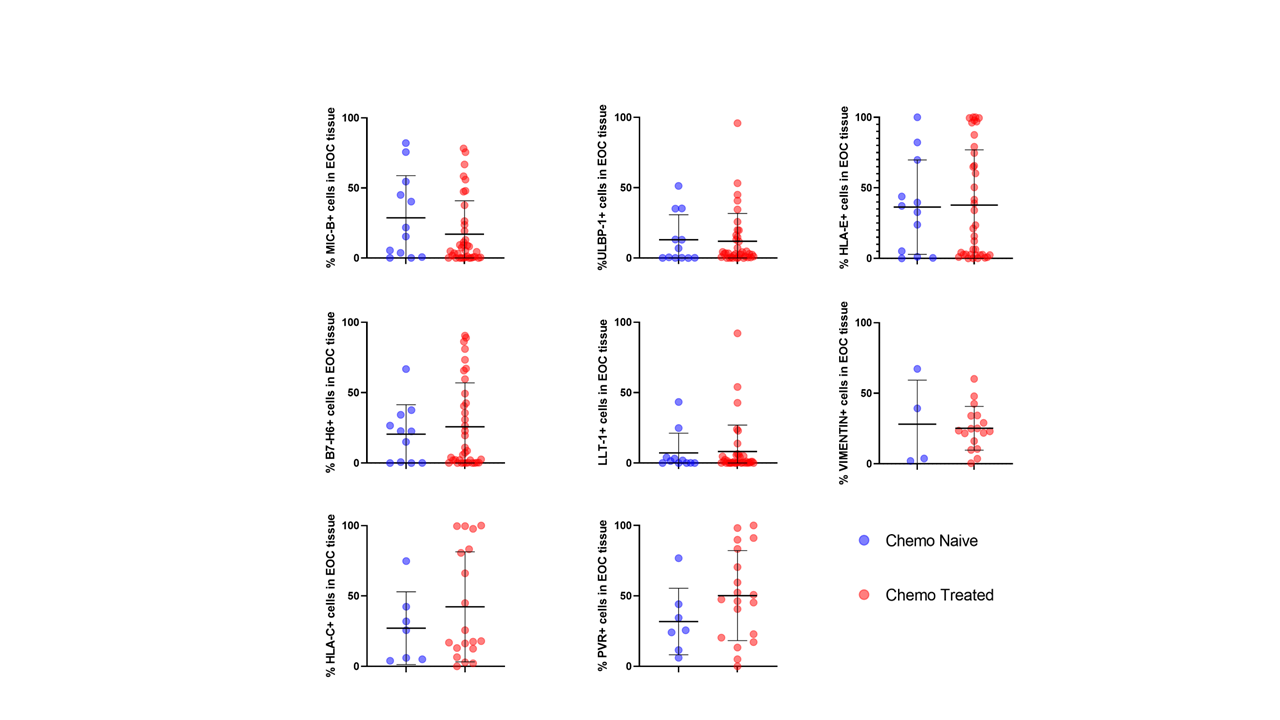

Supplement: Supplementary Figure 7 — Ligand expression profile of EpCAM+ HGSOC tumour cells in primary chemotherapy naïve and treated cohort. [file Image7.tif]

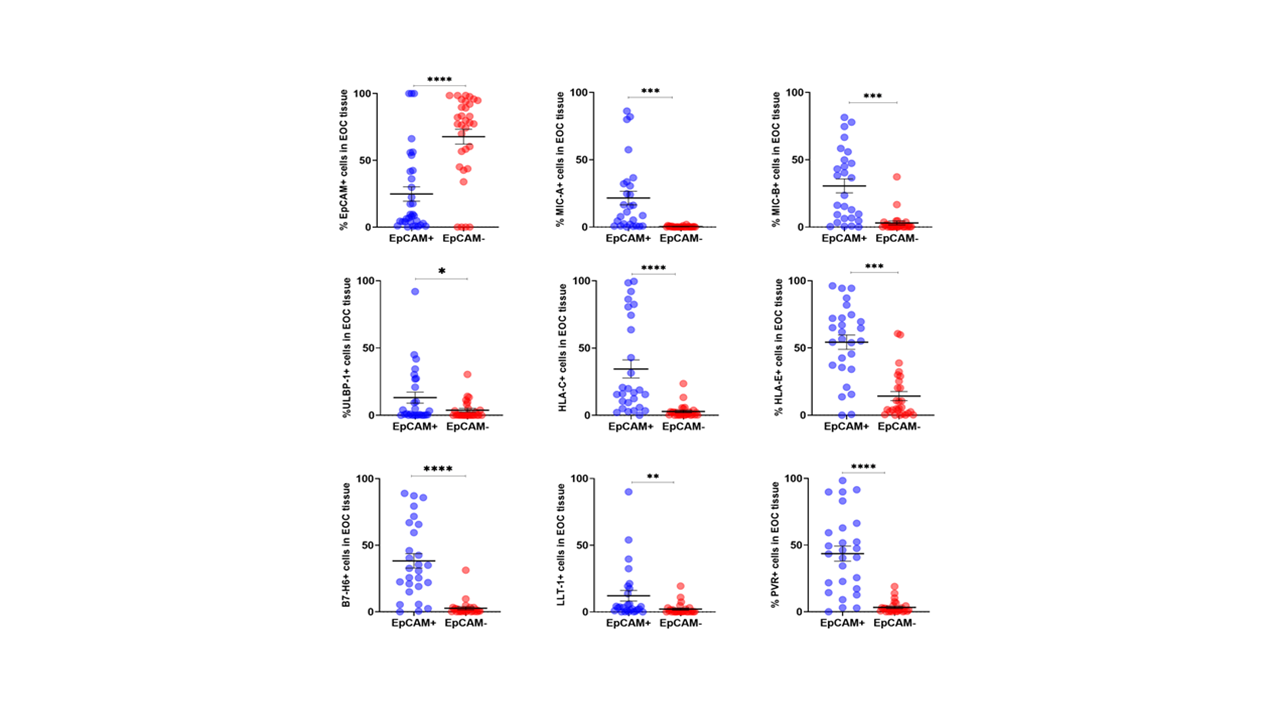

Supplement: Supplementary Figure 8 — Ligands expression profile of EpCAM+ and EpCAM- cells in HGSOC patients *p<0.05; **p<0.01; ***p<0.001; ****p<0.0001. [file Image8.tif]
