# Supplementary material for: Chemotherapy driven alterations in NK cell receptors and ligands in high grade serous ovarian cancer
Source: Front Immunol. 2026 Mar 31;17:1765987. doi: 10.3389/fimmu.2026.1765987 (PMC13076314; doi:10.3389/fimmu.2026.1765987)
Supplement: Supplementary file 9 [file Table1.docx]

| **Patient’s characteristics** | | |
| --- | --- | --- |
| Group | Primary debulking surgery (n=33) | Interval debulking surgery  (n=57) |
| Age median (range) | 51.5(34-74) | 56 (32-78) |
| Chemotherapy  at the time of enrollment | Not done | 3 cycles of chemotherapy (Paclitaxel+Carboplatin) |
| **Stage** | | |
| II | 4 (12.1 %) | 6 (10.5 %) |
| III | 21 (63.6 %) | 42 (73.6 %) |
| IV | 8 (24.2 %) | 9 (15.7 %) |
| **Grade** | | |
| High | 28 (84.8 %) | 57 (100 %) |
| Low | 2 (6.0 %) | 0 (0 %) |
| Unknown | 3 (9.0 %) | 0 (0 %) |
| **Co-morbidity** | | |
| Yes | 17 (51.5 %) | 28 (49.12%) |
| No | 13 (39.3 %) | 26 (45.61 %) |
| Unknown | 3 (0.9 %) | 3 (5.26 %) |
| **Fluid cytology** | | |
| Positive | 18 (54.5 %) | 26 (45.61 %) |
| Negative | 11 (33.3 %) | 29 (50.87 %) |
| Unknown | 4(12.1 %) | 2 (3.5 %) |
| **Lymph node metastasis** | | |
| Positive | 18 (54.5 %) | 23 (40.35 %) |
| Negative | 13 (39.3 %) | 28 (49.12%) |
| Unknown | 2 (6.0 %) | 6 (10.52 %) |
| **CA-125 Median (Range) U/ml** | | |
| Pre-treatment | 503.75 (68.5 - 5111) | 987.5 (29.7-30186) |
| Post-treatment | 14.7(3.9 - 293.7) | 34.23 (3.9-229.68) |

Supplementary Table S1 : Detailed patient characteristics
