# Supplementary material for: Chemotherapy driven alterations in NK cell receptors and ligands in high grade serous ovarian cancer
Source: Front Immunol. 2026 Mar 31;17:1765987. doi: 10.3389/fimmu.2026.1765987 (PMC13076314; doi:10.3389/fimmu.2026.1765987)
Supplement: Supplementary file 10 [file Table2.docx]

Supplementary Tabel 2 : Details of antibodies and reagents

| Panel 1 | Clone |  |  | Panel 3 | Clone | Catalog | RRID |
| --- | --- | --- | --- | --- | --- | --- | --- |
| Fluorescent labeled antibody |  | Catalog | RRID | Fluorescent labeled antibody |  |  |  |
| Vivid dye- BV421 |  |  |  | Vivid-dye BV421 |  |  |  |
| CD45-PECY5.5 | HI30 | 304010 | AB_314398 | EpCAM-BV480 | EBA-1 | 746376 | AB_2743692 |
| CD56-PE | 5.1H11 | 981202 | AB_2715758 | B7-H6-PE | 875001 | FAB7144P | AB_3652285 |
| CD3-PECF594 | UCHT1 | 562280 | AB_11153674 | MICA-AF488 | 159227 | FAB1300G | AB_10891134 |
| NKG2D-APC-CY7 | 1D11 | 320824 | AB_2566660 | LLT-1 APC | 402659 | FAB3480A | AB_2044641 |
| CD161-BV480 | DX12 | 746305 | AB_2743630 | ULBP-1 AF750 | 170818 | FAB1380S | AB_3646362 |
| DNAM-1-BV786 | 11A8 | 752660 | AB_2917644 | MICB-AF594 | 236511 | FAB1599T | AB_3646782 |
| NKp30-BV605 | p30-15 | p30-15 | AB_2738170 | HLA-C-BV605 | DT-9 | 747596 | AB_2744165 |
| NKp46-PerCP-cy5.5 | 9E2 | 3.32E+05 | AB_2561664 | PVR-PerCP/CY5.5 | SKll4 | 337612 | AB_2565536 |
| NKG2A-APC | S19004C | 375107 | AB_2888862 | HLA-E PECY7 | 3D12 | 342602 | AB_1659247 |
|  |  |  |  |  |  |  |  |
| Panel 2 | Clone |  |  |  |  |  |  |
| Fluorescent labeled antibody |  | Catalog | RRID |  |  |  |  |
| Vivid-Dye-BV421 |  |  |  |  |  |  |  |
| CD45-PECY5.5 | HI30 | 304010 | AB_314398 |  |  |  |  |
| CD56-PE | 5.1H11 | 981202 | AB_2715758 |  |  |  |  |
| CD3-PECF594 | UCHT1 | 562280 | AB_11153674 |  |  |  |  |
| NKp44-PECY7 | P44-8 | 325116 | AB_2616754 |  |  |  |  |
| NKG2C-AF488 | 134522 | FAB1381G | AB_3646366 |  |  |  |  |
| KIR2DL1/S1-APC | EB6B | A22332 |  |  |  |  |  |
| KIR3DL1-BV786 | DX9 | 742982 | AB_2741186 |  |  |  |  |
| **Reagents and kits** | | | | | | | |
| Reagents |  | Catalog |  | Reagents |  | Catalog |  |
| EDTA vacutainer |  | 367835 |  | RPMI medium |  | 11875093 |  |
| Sterile surgical blade |  | PL68 |  | PBS |  | 10010023 |  |
| Collagenase type IV |  | 17104019 |  | FACS lysis buffer |  | 349202 |  |
| Trypsin EDTA |  | 25300054 |  | Violet Dead cell stain kit |  | L34964 |  |
| Procartaplex Immuno assay |  | PPX-12 |  | GraphPad Prism 9.0 |  |  |  |
| MIC-A ELISA |  | BMS2302 |  |  |  |  |  |
| MIC-B ELISA |  | BMS2303 |  |  |  |  |  |
| ULBP-1 ELISA |  | EH476RB |  |  |  |  |  |
